# Exon Junction Complex Shapes the Transcriptome by Repressing Recursive Splicing

**DOI:** 10.1016/j.molcel.2018.09.033

**Published:** 2018-11-01

**Authors:** Lorea Blazquez, Warren Emmett, Rupert Faraway, Jose Mario Bello Pineda, Simon Bajew, Andre Gohr, Nejc Haberman, Christopher R. Sibley, Robert K. Bradley, Manuel Irimia, Jernej Ule

**Affiliations:** 1 Department of Neuromuscular Diseases, UCL Queen Square Institute of Neurology, Queen Square, London WC1N 3BG, UK; 2 The Francis Crick Institute, 1 Midland Road, London NW1 1AT, UK; 3 University College London Genetics Institute, Gower Street, London WC1E 6BT, UK; 4 Centre for Genomic Regulation (CRG), The Barcelona Institute of Science and Technology, Dr Aiguader 88, 08003 Barcelona, Spain; 5 Computational Biology Program, Public Health Sciences Division, Fred Hutchinson Cancer Research Center, Seattle, WA 98109, USA; 6 Basic Sciences Division, Fred Hutchinson Cancer Research Center, Seattle, WA 98109, USA; 7 Department of Genome Sciences, University of Washington, Seattle, WA 98195, USA; 8 Medical Scientist Training Program, University of Washington, Seattle, WA 98195, USA

**Keywords:** gene expression, alternative splicing mechanisms, recursive splicing, exon junction complex, RS exon, microexon, microcephaly, neurodevelopmental disorders, evolution

## Abstract

Recursive splicing (RS) starts by defining an “RS-exon,” which is then spliced to the preceding exon, thus creating a recursive 5′ splice site (RS-5ss). Previous studies focused on cryptic RS-exons, and now we find that the exon junction complex (EJC) represses RS of hundreds of annotated, mainly constitutive RS-exons. The core EJC factors, and the peripheral factors PNN and RNPS1, maintain RS-exon inclusion by repressing spliceosomal assembly on RS-5ss. The EJC also blocks 5ss located near exon-exon junctions, thus repressing inclusion of cryptic microexons. The prevalence of annotated RS-exons is high in deuterostomes, while the cryptic RS-exons are more prevalent in *Drosophila*, where EJC appears less capable of repressing RS. Notably, incomplete repression of RS also contributes to physiological alternative splicing of several human RS-exons. Finally, haploinsufficiency of the EJC factor *Magoh* in mice is associated with skipping of RS-exons in the brain, with relevance to the microcephaly phenotype and human diseases.

## Highlights

• Hundreds of RS-exons in human mRNAs reconstitute cryptic 5ss at exon-exon junctions
• EJC represses recursive splicing of RS-exons to preserve transcriptome integrity
• RS contributes to physiological alternative splicing
• EJC-mediated repression is particularly important in deuterostomes and in the brain

## Graphical Abstract

**Figure:**
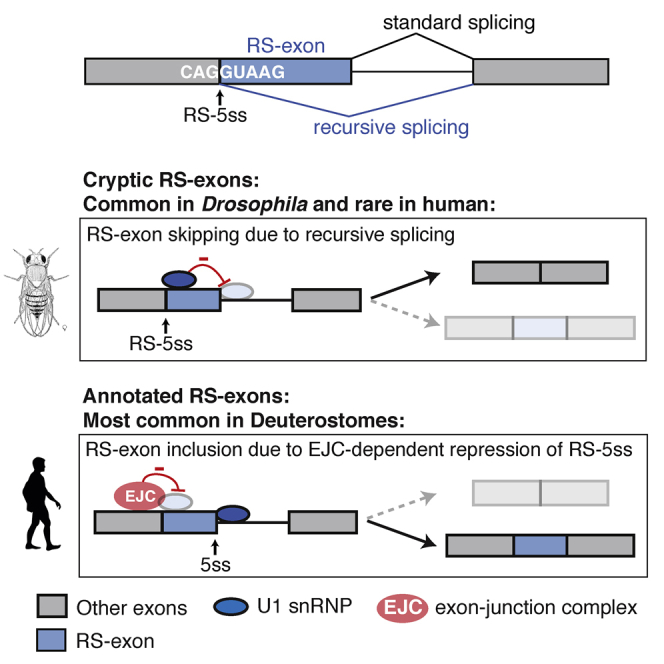

## Teaser

Blazquez et al. demonstrate that hundreds of annotated exons can be skipped from partly spliced transcripts through a mechanism called recursive splicing. Deposition of the exon junction complex represses this mechanism, which is particularly relevant in deuterostomes and the brain, where it can contribute to a microcephaly phenotype and human disease.

## Introduction

**Figure 1:**
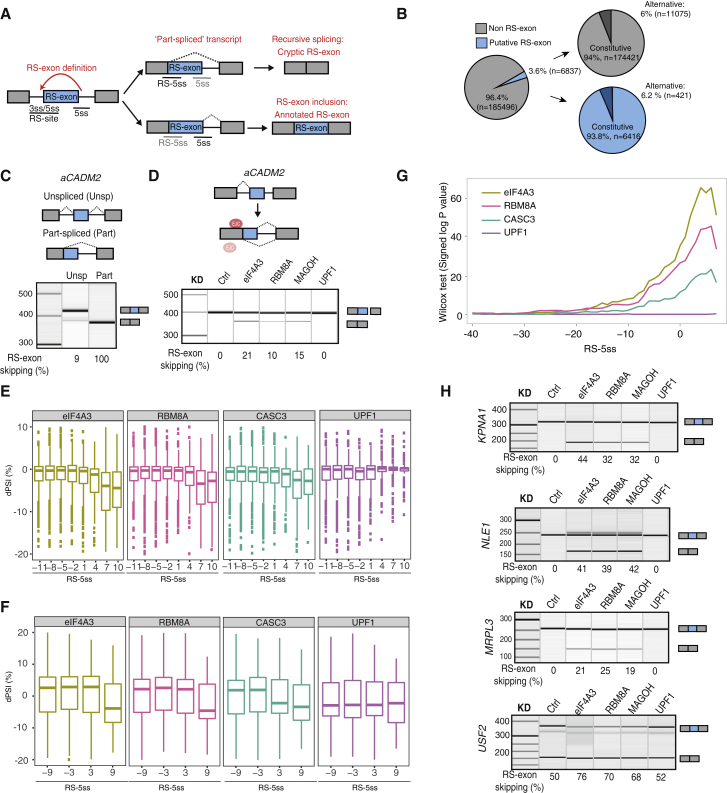
Core EJC Components Promote Inclusion of Putative “RS-Exons” (A) An RS-exon starts with a partial 5ss motif, and after the first step of splicing to the preceding exon, it generates RS-5ss within the part-spliced transcript. The RS-exon will be skipped if the RS-5ss is used for the second step of splicing and included if the canonical 5ss is used. (B) Pie charts show the prevalence of putative RS-exons in human mRNAs according to ENSEMBL GRCh37 annotation. (C) RT-PCR analysis of unspliced and part-spliced reporters derived from the alternative *CADM2* (*aCADM2*) isoform after transient transfection into HeLa cells. (D) *aCADM2* unspliced reporter was stably integrated into the genome of HeLa cells, and the splicing pattern of the RS-exon was analyzed by RT-PCR after eIF4A3, RBM8A, MAGOH, and UPF1 KD. (E) Boxplots show the difference in percentage spliced in (dPSI) of highly included exons (PSI > 90%) after KD of eIF4A3, RBM8A, CASC3, or UPF1. Exons are binned by their RS-5ss score, and dPSI for each bin is calculated by subtracting the PSI in the control experiment to each KD. The RS-5ss values on the x axis indicate the midpoint of each group. Negative dPSI values indicate increased exon skipping upon KD. (F) Same as (E), but for alternative exons with a PSI < 90%. (G) The statistical significance of RS-exon skipping is performed by dividing constitutive RS-exons (PSI >0.98) into two groups based on a RS-5ss score threshold, analyzing the differences in dPSI values between the two groups, and calculating a signed p-value by testing for a skew in dPSI values between the two groups using the Wilcoxon rank-sum test. The analysis is done at multiple thresholds, from −40 to 8. (H) RT-PCR analysis of RS-exon splicing pattern after KD of EJC core factors or UPF1. Results shown in (C), (D), and (H) derive from a minimum of 3 independent experiments performed in HeLa cells.

Alternative splicing (AS) is regulated by many proteins that bind to the nascent RNA to modulate the initial step of exon definition (Jangi and Sharp, 2014). However, less is known about the role of proteins binding to spliced RNA, which could regulate a two-step splicing process, such as recursive splicing (RS) (Hatton et al., 1998, Sibley et al., 2015). RS starts by definition of an “RS-exon,” which contains a partial 5′ splice-site (5ss) motif at its beginning. When the RS-exon is spliced to its preceding exon, it reconstitutes a new 5ss (RS-5ss) at the exon-exon junction of the part-spliced transcript (Figure 1A). In the second step of RS, use of the RS-5ss leads to skipping the RS-exon along with the downstream intron. Transcriptomic studies of RS-exons in vertebrates and *Drosophila* have so far focused on cryptic exons, which are removed without a trace due to the highly efficient use of their RS-5ss (Joseph et al., 2018, Sibley et al., 2015). Nevertheless, we have identified very rare isoforms in a few genes where the RS-exons are included and showed that the inclusion of the RS-exon is determined by competition between the RS-5ss and the downstream 5ss of the RS-exon (Figure 1A) (Sibley et al., 2015). However, the factors that could bind to the part-spliced transcript to regulate inclusion of RS-exons remained unknown.

The exon junction complex (EJC) is deposited on the spliced transcript ∼20–24 nt upstream of each exon-exon junction. The EJC core is composed of eIF4A3 RNA helicase and MAGOH and RBM8A that are deposited as a heterodimer that stabilizes the binding of eIF4A3 by inhibiting its ATPase activity (Le Hir et al., 2016). The EJC has multiple roles in post-splicing events, such as mRNA transport, translation, and surveillance by nonsense-mediated decay (NMD) (Le Hir et al., 2016). It also promotes inclusion of specific exons in *Drosophila* (Ashton-Beaucage et al., 2010, Roignant and Treisman, 2010) and humans (Michelle et al., 2012, Wang et al., 2014), and the underlying mechanism was proposed to involve enhanced spliceosome recruitment to nearby splice sites or a change in the speed of RNA polymerase II (PolII) elongation (Le Hir et al., 2016). eIF4A3 is deposited to the 5′ exon during the splicing reaction via interactions with the spliceosomal protein CWC22 before the exon-exon junction is fully formed (Le Hir et al., 2016). Due to its early recruitment, the EJC thus has the capacity to affect the second step of any two-step splicing process.

We examined the role of the EJC in the regulation of RS. We find that the EJC blocks recognition of RS-5ss to promote inclusion of “annotated RS-exons.” Knockdown (KD) of core EJC factors, and the peripheral factors RNPS1 and PNN, leads to widespread skipping of annotated RS-exons via RS. We show that depletion of eIF4A3 increases the assembly of spliceosome on RS-5ss, as demonstrated by crosslinking and immunoprecipitation (iCLIP) with the core spliceosomal protein PRPF8. Moreover, EJC deposition also impedes the recognition of 5ss located downstream of exon-exon junctions, thereby repressing inclusion of short exons also known as microexons. The function of the EJC is required for efficient inclusion of annotated RS-exons in the mouse brain, as *Magoh* haploinsufficient brain shows RS-exon skipping in genes that could contribute to the microcephaly phenotype. Notably, analysis of PRPF8 iCLIP and intron lariats shows that incomplete EJC-mediated repression of RS also represents a mechanism for physiological AS of several human RS-exons. Evolutionary comparison of sequence features at exon-exon junctions shows that the number of annotated RS-exons increased in the deuterostome clade, which contrasts the large number of cryptic RS-exons in *Drosophila*. Taken together, we show that the EJC is required to repress RS-5ss in mammalian mRNAs, which is required for high inclusion of hundreds of annotated RS-exons, thus potently shaping and regulating the mammalian transcriptome.

## Results

### Core EJC Components Promote Inclusion of Putative RS-Exons, which Are Abundant in the Transcriptome

We consider any exon that reconstitutes a 5ss (RS-5ss) at the preceding exon-exon junction in the part-spliced transcript as a putative RS-exon (Figure 1A). To assess the prevalence of such putative RS-exons in human transcripts, we examined the sequence at exon-exon junctions that precede all annotated human exons using MaxEntScan software (Yeo and Burge, 2004). First, we defined a MaxEntScan threshold for putative RS-exons by analyzing the distribution of canonical 5ss scores at exon-intron junctions (Figure S1A). Next, we calculated the MaxEntScan score at every exon-exon junction (RS-5ss) and defined putative RS-exons as those with a RS-5ss that exceeded a given threshold. Using thresholds that detect 75% to 95% of canonical 5′ss, the proportion of exons in the transcriptome that would be classified as putative RS-exons varied between 1.7% and 4.9%, respectively (Figure S1B). For consistency, all further analyses in this manuscript used a threshold that detects 90% of human canonical 5ss, which has an associated MaxEntScan score of 5.52. According to this threshold, 3.6% of human exons are putative RS-exons (n = 6,837; Figure 1B), and 21.7% of genes (n = 4,900) and 16.2% of transcripts (n = 5,295) contain at least one putative RS-exon (Figure S1C).

The vast majority of putative RS-exons (93.8%, n = 6,416) are constitutive according to ENSEMBL (Figure 1B), which raises the question of why the RS-5ss of these exons are not used. To understand this question, we used a splicing reporter from the rare isoform of *CADM2* gene (*aCADM2*), which retains the RS-exon (Sibley et al., 2015). We designed two versions of the reporter: the unspliced version contains both introns flanking the RS-exon, whereas the part-spliced version lacks the first intron. Strikingly, while the RS-exon is almost completely included in the unspliced reporter, it is fully skipped in the part-spliced reporter (Figures 1C and S1D). Thus, the first splicing step that removes the preceding intron is crucial for RS-exon inclusion in *aCADM2*, indicating that it might deposit factors such as EJC onto the part-spliced transcript that repress the RS-5ss.

To test if EJC indeed contributes to *aCADM2* RS-exon inclusion, we stably integrated the *aCADM2* unspliced reporter into HeLa cells and monitored the effect of eIF4A3, RBM8A, and MAGOH KD. KD of either MAGOH or RBM8A led to depletion of both factors, probably because they act as a heterodimer (Figure S1E). Notably, KD of any of the core EJC factors increased the skipping of the *aCADM2* RS exon (Figures 1D and S1F). As control, we knocked down UPF1, which is required for NMD, but did not affect RS-exon inclusion (Figures 1D and S1F). Thus, the changes in the isoform ratio upon EJC KD are not a side effect of disturbing the NMD pathway.

Given the prevalence of putative RS-exons in the human transcriptome (Figure 1B), we reanalyzed previously published RNA sequencing (RNA-seq) data from HeLa cells upon KD of EJC factors to assess the broader effects on RS-exons. Specifically, we plotted the difference in percentage spliced in (dPSI) in KD compared to control for highly included (PSI > 90%) or alternative exons (PSI < 90%) according to the strength of their RS-5ss (Figures 1E and 1F). dPSI values were grouped into equal sized bins based on their RS-5ss scores, thereby avoiding any initial assumptions in regard to the MaxEntScan score of a valid 5ss. A negative dPSI value indicated more skipping, whereas a positive value denoted more inclusion upon KD. Remarkably, most exons with increased skipping upon KD of EJC factors (negative dPSI values) were those with highest RS-5ss scores. Independent analysis with the H-bond algorithm, an alternative metric of 5ss strength (Freund et al., 2003), gave similar results (data not shown). To assess if the trend toward exon skipping with the increased RS-5ss score is statistically significant, we divided all constitutive exons (PSI > 98) into two groups based on their RS-5ss score and tested for a skew in dPSI values between the two groups by calculating a signed p value with the Wilcoxon rank-sum test. By performing this analysis with multiple thresholds, we confirmed that the trend toward exon skipping as the RS-5ss score increases is highly significant (Figure 1G). RT-PCR validated several annotated RS-exons as sensitive to eIF4A3, RBM8A, and MAGOH KD, but not UPF1 (Figures 1H, S1G, and S1H). This confirms that all of the core EJC components are crucial to inhibit the skipping of hundreds of putative RS-exons.

### PNN and RNPS1 Contribute to the Inclusion of Annotated RS-Exons

**Figure 2:**
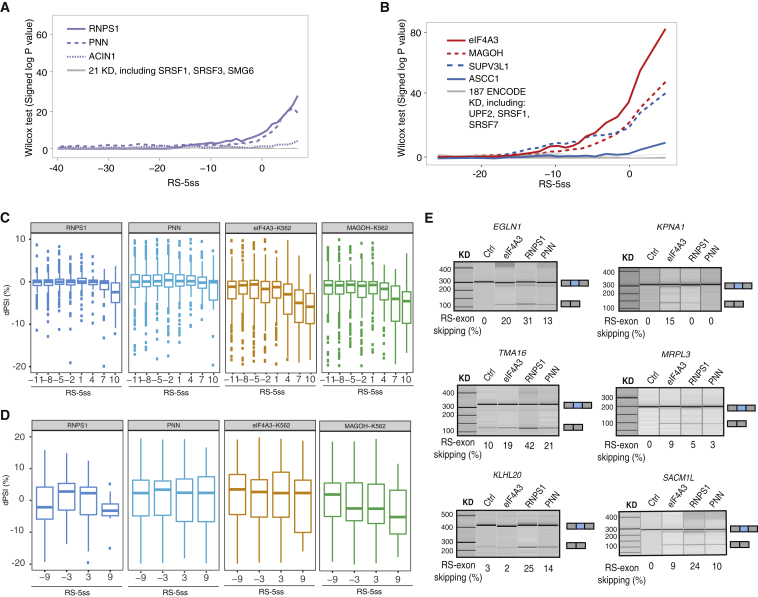
PNN and RNPS1 Contribute to the Inclusion of “Annotated RS-Exons” (A) The statistical significance of RS-exon skipping upon KD of 28 RBPs is performed by analysis of public RNA-seq data as explained in Figure 1G. Peripheral EJC factors are marked in purple, and experiments that had no effect are marked in gray. (B) The statistical significance of RS-exon skipping upon KD of 191 RBPs calculated by analysis of ENCODE consortium RNA-seq, as in (A). Core EJC components are marked in red, new factors that had an effect are marked in blue, and KD experiments that had no effect are marked in gray. (C) Distribution of dPSI after KD of different RBPs is shown for highly included exons in control (PSI > 90%), as explained in Figure 1E. (D) Same as (C), but for alternative exons with PSI < 90%. (E) RT-PCR validation of RS-exon skipping after KD of eIF4A3, RNPS1, or PNN (n = 3 independent experiments for *KLHL20* and *TMA16*; n = 2 for *EGLN1*, *KPNA1*, *MRPL3*, and *SACM1L*).

For insights into additional factors that contribute to inclusion of RS-exons, we analyzed all available RNA-seq data from studies that included KD of at least one previously reported EJC-associated factor. Then we examined if there is a significant trend for skipping of constitutive exons with high RS-5ss. KD of EJC peripheral components RNPS1, PNN, and ACIN1 showed significant effects, although the effect of ACIN1 was very weak (Figure 2A). We also analyzed RNA-seq data from the ENCODE Consortium that includes KD of 191 RNA binding proteins (RBPs) and identified significant effects for eIF4A3, MAGOH, SUPV3L1, and ASCC1 KD (Figure 2B). Notably, KD of other factors that were reported to associate with the EJC, such as SRSF1 and SRSF3 serine/arginine-rich (SR) proteins (Singh et al., 2012) or components of the NMD machinery, did not show any effect, indicating that they do not play an important role in the EJC assembly on part-spliced transcripts.

To further visualize the splicing changes, we plotted the KD-induced change in PSI (ΔPSI) for groups of exons with similar RS-5ss scores (Figure 2C). Highly included annotated RS-exons generally increased skipping by 4%–10% upon KD of core EJC components and 3%–6% upon KD of RNPS1 and PNN. Increased skipping of alternative annotated RS-exons was also evident, even though the effect was milder and most apparent for RNPS1 and MAGOH (Figure 2D). RT-PCR analysis after KD of eIF4A3, RNPS1, or PNN in HeLa cells indicated that each annotated RS-exon has a unique sensitivity profile to the depletion of different EJC factors. Some RS-exons (i.e., KPNA1) do not require EJC peripheral factors for their inclusion, whereas others are more sensitive to peripheral factors than to core factors (Figures 2E and S2C). Analysis of ENCODE data uncovered two unexpected factors, SUVP3L1 and ASCC1 (Figure 2B). We noticed that the short hairpin RNA (shRNA) sequence used for SUVP3L1 KD is highly complementary to eIF4A3 mRNA and results in a strong downregulation of eIF4A3 (Figure S2F). When we used a different small interfering RNA (siRNA) sequence for SUVP3L1 KD, we could not validate any of the splicing changes detected in ENCODE RNA-seq data (Figure S2G), indicating that the changes seen in ENCODE data were most likely caused by off-target effect of SUVP3L1 shRNA. In the case of ASCC1 KD, the trend toward RS-exon skipping was minor and could also not be validated (Figures 2B and S2E). Taken together, our results show that the core and peripheral EJC factors are required to repress skipping of both constitutive and alternative annotated RS-exons, which supports previous biochemical findings that RNPS1 and PNN associate at the earliest stages of EJC assembly in the nucleus (Le Hir et al., 2016). Variable sensitivities of RS-exons for KD of different factors also agree with previous findings that the importance of each factor in EJC assembly could vary between exon-exon junctions (Saulière et al., 2012, Singh et al., 2012).

### Skipping of Annotated RS-Exons upon EJC KD Results from RS

**Figure 3:**
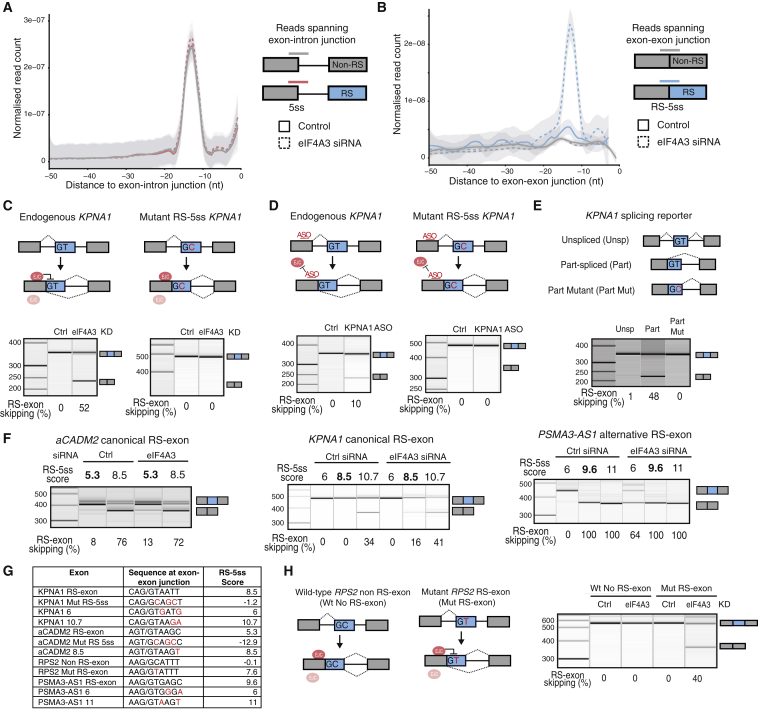
Skipping of Annotated RS-Exons upon EJC KD Results from Recursive Splicing (A) The count of PRPF8 iCLIP reads that identify crosslinks at each nucleotide upstream of exon-intron junctions is normalized by the total number of evaluated junctions (RS = 4,631; non-RS = 130,410) and the total number of crosslinks in each experiment. HeLa cells treated with either control or eIF4A3 siRNAs were used for iCLIP (n = 4 per group, 2 independent experiments). Reads upstream of RS-exons and non RS-exons are plotted in orange and gray, respectively. The data were smoothed using locally weighted scatterplot smoothing (LOESS). Shaded regions represent 95% confidence intervals. (B) Density plot as in (A), but assessing PRPF8 iCLIP upstream of exon-exon junctions. Crosslinks upstream of RS-exons or non RS-exons are plotted in blue or gray, respectively. (C) *KPNA1* unspliced reporter with a mutant RS-5ss was stably integrated into HeLa cells, and the splicing pattern of endogenous or mutant RS-exon was analyzed by RT-PCR after eIF4A3 KD. (D) Same analysis as in (C), after transfecting cells with an antisense oligonucleotide complementary to the EJC deposition site. (E) RT-PCR analysis of wild-type and mutant unspliced and part-spliced reporters derived from the *KPNA1* gene after their transient transfection into HeLa cells. (F) RT-PCR analysis of RS-exon splicing pattern after transient transfection of wild-type or mutant *aCADM2*, *KPNA1*, and *PSMA3-AS1* reporters into HeLa cells treated with control or eIF4A3 siRNAs. The original RS-5ss score is indicated in bold, and the scores after mutation are in regular font. (G) Nucleotide sequences at the exon-exon junctions and their associated RS-5ss scores for the splicing reporters used in (C), (F), and (H). The nucleotides before and after the slash sign correspond to the last 3 nt of the preceding exon and the first 6 nt of the exon under study. Mutations are indicated in red. (H) Wild-type and mutant splicing reporters derived from *RPS2* gene were transiently transfected into HeLa cells, and their splicing pattern was analyzed by RT-PCR after control or eIF4A3 KD. Results shown in (C)–(F) and (H) derive from a minimum of 3 independent replicates.

To validate RS as the mechanism responsible for skipping of annotated RS-exons, we performed PRPF8 iCLIP in eIF4A3 KD and control conditions. PRPF8 is an integral component of the U5 small nuclear ribonucleoprotein particle (snRNP) and binds upstream of 5ss (Teigelkamp et al., 1995). Since iCLIP cDNAs generally truncate at the crosslink sites of RBPs (Haberman et al., 2017), we expected that the ungapped PRPF8 iCLIP reads could be mapped across exon-intron junctions and gapped reads across exon-exon junctions to validate spliceosome assembly at canonical 5ss and RS-5ss, respectively. Indeed, the ungapped reads identified a sharp PRPF8 crosslinking peak 12–14 nt upstream of 5ss (Figure 3A). The peak did not change upon eIF4A3 KD, demonstrating that eIF4A3 has no general effect on canonical 5ss recognition (Figure 3A). Strikingly, gapped reads also identified a PRPF8 crosslinking peak 12–14 nt upstream of exon-exon junctions that precede RS-exons (Figure 3B). This peak was dramatically increased upon eIF4A3 KD, with no change at non-RS exons, confirming that eIF4A3 blocks spliceosome assembly on RS-5ss across the transcriptome, thereby blocking RS.

PRPF8 crosslinking also confirmed that RNA-seq is generally appropriate to classify RS-exons based on their sensitivity to EJC perturbation, since increased PRPF8 crosslinking is most apparent at EJC-sensitive RS-exons, which were defined by RNA-seq (Figure S3A). This sensitivity cannot be explained by the sequence of the RS-5ss, since both sensitive and insensitive RS-exons have similar MaxEntScan scores (data not shown). Next, we asked if differences in EJC assembly might explain the differential sensitivity. For this, we turned to the previously published iCLIP data for EJC components (Hauer et al., 2016) to compare crosslinking of EJC components at exon-exon junctions. However, no differential EJC binding was observed between the sensitive and insensitive RS-exons (Figure S3B). Finally, we asked if the order of intron removal might play a role (Kim et al., 2017) by examining paired-end RNA-seq reads that map to both ends of annotated RS-exons. If one crossed to the preceding exon and the other crossed to the downstream intron, then the read pair was considered as evidence for the upstream intron being spliced first (co-transcriptional splicing), whereas the reciprocal scenario was considered as evidence for the downstream intron being spliced first (Figure S3C). This analysis indicates that EJC-sensitive RS-exons more often follow a co-transcriptional splicing order when compared to non-sensitive RS-exons. Moreover, EJC-sensitive RS-exons tend to have a stronger 3ss and weaker 5ss, which could also explain why the upstream intron tends to be spliced first (Figure S3C). It is crucial for efficient RS that the upstream intron is spliced first in order to form the RS-5ss that can be used for the second splicing event. We conclude that the annotated RS-exons need to have an asymmetric splicing pattern to be capable of RS upon EJC KD.

We selected several annotated RS-exons for further mechanistic studies with minigene reporters. First, we confirmed that RS-exons require the intact RS-5ss to be efficiently skipped upon eIF4A3 KD, since stable cell lines expressing *KPNA1* and *aCADM*2 unspliced reporters with point mutations at the beginning of their RS-exons remain constitutive (Figures 3C, S3D, and S3E). Second, we blocked EJC binding to the exon preceding *KPNA1* RS-exon using an antisense oligonucleotide (*KPNA1* ASO) complementary to the region where the EJC is deposited. *KPNA1* ASO transfection increased skipping of *KPNA1* RS-exon from the endogenous gene, similar to the effect of eiF4A3 KD. In contrast, *KPNA1* ASO did not promote skipping of *KPNA1* RS-5ss mutant exon (Figures 3D and S3F). Finally, as in a*CADM2*, *KPNA1* reporters that correspond to the sequence produced at the part-spliced stage showed RS-exon skipping, which was dependent on the presence of the intact RS-5ss (Figures 3E, S3G, and S3H).

We have previously shown that efficient RS of cryptic RS-exons requires strong RS-5ss that can outcompete the downstream 5ss of the RS-exon (Sibley et al., 2015). In agreement, increasing the RS-5ss score in *aCADM2* and *KPNA1* RS-exons induced their skipping even in the presence of the EJC (Figures 3F and S3I). In a reciprocal experiment, decreasing the RS-5ss score in constitutive RS-exons made the constitutive *KPNA1* RS-exon completely insensitive to EJC depletion, while the *PSMA3-AS1* RS-exon becomes less sensitive (Figures 3F and S3I). Both RS-exons shared the same RS-5ss score (Figure 3G), but their variable sensitivity could result from differences in their RS-5ss sequences, the structure of RNA, or other RBPs that bind nearby to modulate U1 snRNP recognition. Importantly, a GC-to-GT mutation at the beginning of the *RPS2* non RS-exon was sufficient to turn it into a RS-exon sensitive to eIF4A3 KD (Figures 3H and S3J). In conclusion, it is clear the strength of RS-5ss determines whether an RS-exon is constitutive or alternative in the presence of EJC and whether it becomes skipped upon depletion of the EJC. Altogether, the PRPF8 iCLIP and minigene experiments demonstrate that the increased skipping of annotated RS-exons upon EJC KD is a result of RS.

### EJC Assembly Is Required to Stabilize eIF4A3 Binding to Block RS

**Figure 4:**
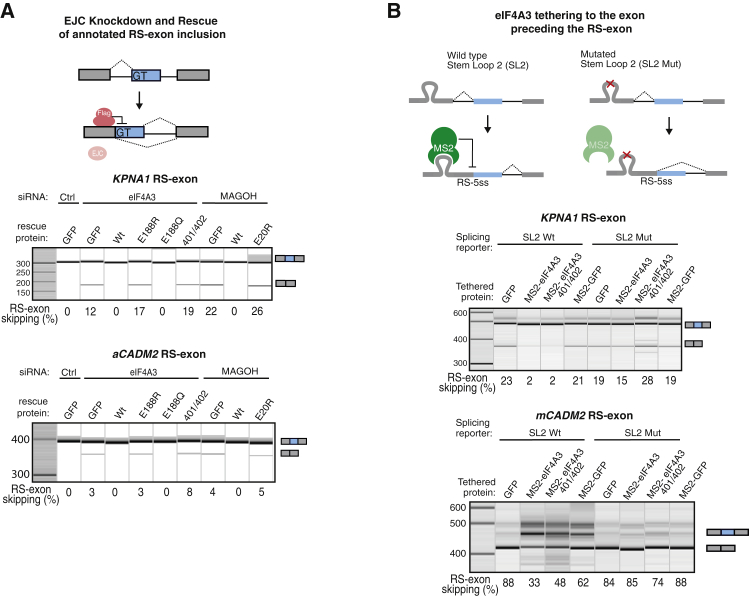
Stable EJC Deposition Is Required to Block Recursive Splicing (A) KD of EJC components was rescued with siRNA-resistant FLAG-tagged counterparts. The splicing pattern of endogenous *KPNA1* or stably integrated *aCADM2* RS-exons was monitored by RT-PCR. (B) Wild-type or mutant SL2 sequence was inserted into *KPNA1* and *mCADM2* splicing reporters at the expected EJC deposition site, and the splicing pattern was monitored by RT-PCR after co-transfection of SL2 reporters and plasmids expressing the indicated MS2-tagged proteins. A minimum of 3 independent replicates was performed in HeLa (A) or 293 (B) cells.

We next asked if assembly of the complete EJC is crucial to inhibit RS. We first established siRNA-resistant FLAG-tagged eIF4A3 or MAGOH constructs, which efficiently rescued a*CADM2* and *KPNA1* RS-exon inclusion when expressed at similar levels to their endogenous counterparts upon EJC KD (Figure S4A). We then used three mutants (eIF4A3 E188R, eIF4A3 401/402, and MAGOH E20R) previously reported to be unable to assemble the EJC (Gehring et al., 2009), which could not rescue the function of the endogenous proteins (Figures 4A, S4B, and S4C). Interestingly, expression of these mutants also induced RS-exon skipping under control conditions (Figures S4D and S4E), in agreement with their previously described dominant-negative function (Wang et al., 2014). On the contrary, the eIF4A3 E188Q mutant, which inactivates the ATPase activity of eIF4A3 while remaining capable of EJC assembly (Shibuya et al., 2006), rescued the function of the endogenous protein (Figure 4A, S4B, and S4C). Taken together, this confirms that stable assembly of the endogenous EJC is required to repress RS of annotated RS-exons.

MAGOH-RBM8A proteins stabilize eIF4A3 binding to RNA by inhibiting its ATPase activity (Ballut et al., 2005, Gehring et al., 2009). Thus, we asked if eIF4A3 could repress RS when stably tethered to RNA, even if it is incapable of assembling an EJC. We designed a *KPNA1* unspliced tethering reporter in which the EJC deposition site was substituted by one MS2 bacteriophage stem loop 2 (SL2) and co-expressed the MS2 coat protein (MS2-CP) fused to either wild-type eIF4A3 or eIF4A3 401/402 mutant, which does not assemble the EJC. Both wild-type and eIF4A3 401/402 mutant efficiently repressed *KPNA1* RS-exon skipping, whereas MS2-GFP could not (Figures 4B and S4G). As a negative control we designed a *KPNA1* SL2 Mut splicing reporter, where SL2 mutation prevents tethering of the MS2-CP, to confirm that MS2-eIF4A3 is incapable of repression without tethering (Figures 4B and S4G). Notably, tethering MS2-eIF4A3 upstream of the exon-exon junction of a cryptic RS-exon in the *CADM2* gene (*mCADM2*) that normally undergoes very efficient RS (Sibley et al., 2015) also turned it into a highly included exon (Figures 4B and S4H). Thus, endogenous EJC assembly is crucial to repress RS, while the tethered assembly-deficient eIF4A3 401/402 mutant is efficient on its own. This observation demonstrates that the role of endogenous EJC assembly is to stabilize eIF4A3 binding on the part-spliced transcript in order to repress RS.

### EJC Depletion Leads to Inclusion of Cryptic Microexons

**Figure 5:**
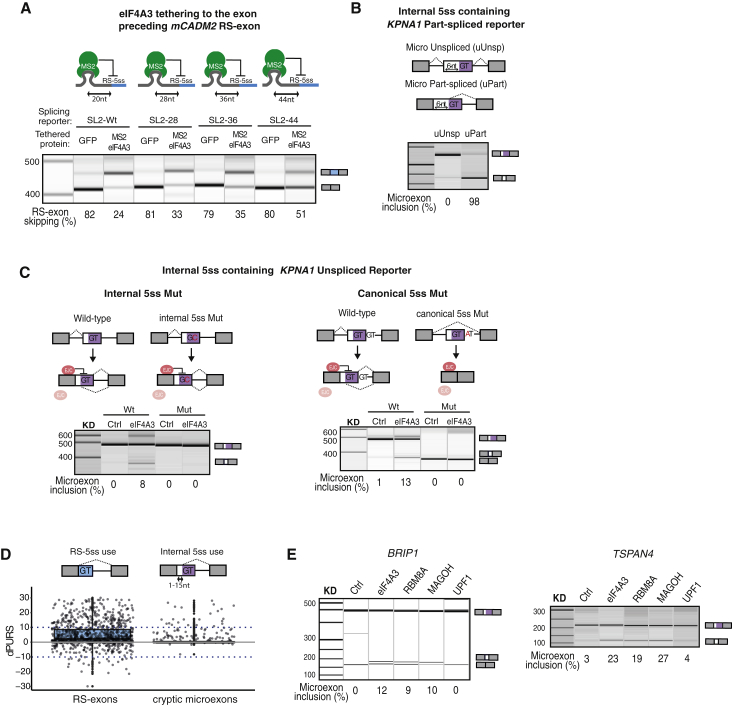
EJC Depletion Leads to Inclusion of Cryptic Microexons (A) The SL2 in *mCADM2* splicing reporter was moved upstream of the exon-exon junction as indicated, and the RS-exon splicing pattern was analyzed after co-transfection of SL2 reporters and plasmids expressing MS2-eIF4A3 or GFP proteins. (B) Six nucleotides (GCACAG) were added at the beginning of the *KPNA1* RS-exon to move the RS site to an internal 5ss, thus creating a *KPNA1* microexon reporter. Unspliced and part-spliced version of the reporters were transfected, and their splicing pattern was analyzed by RT-PCR. (C) The internal or canonical 5ss within *KPNA1* microexon unspliced reporter were mutated, and the splicing pattern was analyzed by RT-PCR after transfection into HeLa cells treated with control or eIF4A3 siRNAs. (D) The difference in the use of RS-5ss or internal 5ss in EJC KD compared to control (dPURS) is shown in HeLa cells. Cryptic microexons result from the use of an internal 5ss that is located within the first 15 nt of a longer annotated exon. Positive dPURS values indicate increased RS-exon skipping or increased inclusion of cryptic microexons. (E) RT-PCR validation of 2 microexon inclusion events identified in (D) after KD of EJC core factors or UPF1. Results shown in (A)–(C) and (E) derive from a minimum of 3 independent replicates performed in HeLa cells.

To understand if the distance between eIF4A3 binding and RS-5ss affects RS repression, we moved the SL2 sequence in the *mCADM2* splicing reporter from 20 nt to 28, 36, or 44 nt upstream of the exon-exon junction (Figure 5A). Co-expression of MS2-eIF4A3 led to *mCADM2* cryptic RS-exon inclusion in all cases, although increasing the distance gradually diminished the repressive capacity of eIF4A3 (Figures 5A and S5A). Therefore, we hypothesized that the endogenous EJC could repress a cryptic splice site also if it is located a few nucleotides away from the exon-exon junction. For example, if an internal 5ss was present within the first 15 nt of a long exon, then its recursive recognition would result in inclusion of a short exon that could be considered a microexon (defined as any exon shorter than 15 nt; Irimia et al., 2014). To test this hypothesis, we shifted the position of the RS-5ss in *KPNA1* 6 nt downstream of the exon-exon junction. In the unspliced reporter, this internal 5ss was repressed, but in the part-spliced reporter, it was used, leading to inclusion of a 6-nt microexon (Figures 5B and S5B). In agreement, eIF4A3 KD also increased the use of internal cryptic 5ss and led to microexon inclusion (Figures 5C and S5C). This demonstrates that EJC recruitment through the first step of splicing is crucial to not only prevent RS-5ss but also repress other cryptic 5ss’s located close to the exon-exon junction.

According to the standard model of splicing regulation, competition between alternative 5ss takes place during exon definition in the pre-mRNA. To test if this model could explain our findings, we generated splicing reporters in which we mutated either the internal or the canonical 5ss of the regulated *KPNA1* exon. As expected, the microexon was no longer included after eIF4A3 KD when the internal 5ss was mutated. Importantly, mutation of canonical 5ss did not increase the use of internal 5ss and microexon inclusion (Figures 5C and S5C), which contradicts the standard model. Instead, it led to whole-exon skipping both in control and eIF4A3-depleted cells. This is consistent with the two-step model of splicing regulation (Figure 1A), where the canonical 5ss is needed for exon definition that initiates the first step of splicing and competition between the two 5ss takes place only in the part-spliced transcript to determine whether the microexon or the full exon will be chosen during the second step of splicing. When EJC deposition inhibits recognition of the internal 5ss, inclusion of the cryptic microexon is repressed, thus ensuring that the annotated exon is fully included.

In order to understand the global role of the EJC in the repression of cryptic microexons hosted by annotated exons, we first identified exons expressed in HeLa cells that either reconstitute a 5ss exactly at the exon-exon junction (putative RS-exons, n = 1,105) or up to 15 nt from the beginning of the exons (putative cryptic microexons, n = 545). We analyzed the splicing pattern of RS-exons and cryptic microexons upon EJC depletion and measured the difference in the use of RS-5ss or internal 5ss in EJC KD compared to control (dPURS). In RS-exons, a positive dPURS indicates increased skipping due to use of RS-5ss, whereas in cryptic microexons, it indicates increased inclusion due to use of the internal 5ss. eIF4A3 KD led to increased skipping in 26.8% of RS-exons, as expected from previous results, but also to inclusion of 5.3% of cryptic microexons (dPURS ≥ 10) (Figure 5D). Interestingly, the distribution of internal 5ss MaxEntScan scores in cryptic microexons was very similar to the distribution of RS-5ss MaxEntScan scores in RS-exons (Figure S5D). RT-PCR analysis validated the inclusion for two of these cryptic microexons in HeLa cells depleted of core EJC factors, but not UPF1 (Figures 5E and S5E).

### EJC-Mediated Repression of RS in the Brain

**Figure 6:**
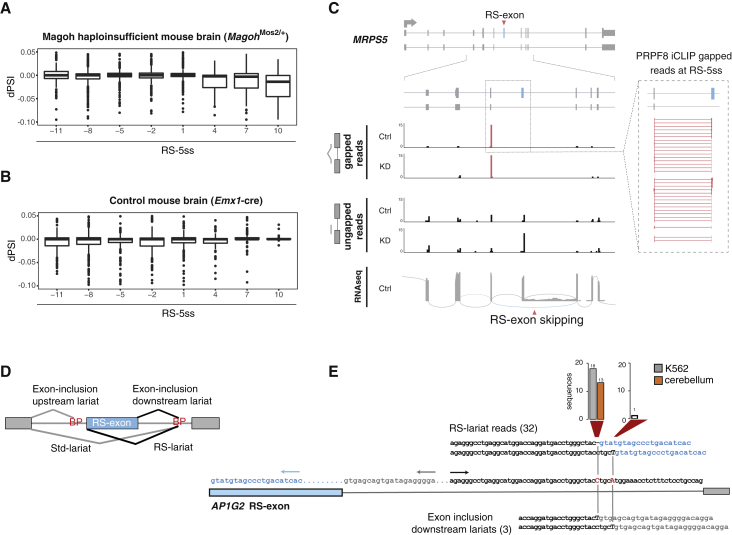
EJC-Mediated Repression of Recursive Splicing in the Brain, and Physiologic Recursive Splicing (A) The dPSI of exons highly included in wild-type mouse brain (PSI > 90%) is shown after comparison with *Magoh* haploinsufficient mouse brain as in Figure 1E. (B) Same as (A), but comparing control *Emx1-cre* and wild-type mouse brain. (C) A schematic of the *MRPS5* gene with the RS-exon highlighted in blue. Below, PRPF8 iCLIP crosslinking is shown, as identified by either ungapped or gapped reads in control or eIF4A3 KD cells (4 replicates are summed up per condition). Crosslinks upstream of junctions involving the RS-exon are shown in red, and corresponding reads are zoomed into on the right. Further below, a Sashimi plot shows RNA-seq evidence of RS-exon skipping. (D) The types of possible lariats associated with RS-exon splicing are named. In black are the lariats that were detected for the *AP1G2* gene as described in (E). (E) Sequence of the RS-exon in the *AP1G2* gene and its downstream intron. BP annotations based on lariat sequencing are highlighted in red. Arrows indicate primers used to interrogate lariats shown in black in (D). Above, sequences and number of reads supporting RS-lariats (inverted alignment) with alternative BP annotations in cerebellum and K562 cells are shown. Below, sequences and number of reads supporting exon inclusion downstream lariats with alternative BP annotations are shown.

Mutations and copy-number variations in *RBM8A* and *EIF4A3* genes are associated with neurodevelopmental phenotypes (Bartkowska et al., 2018). Moreover, haploinsufficiency of *Rbm8a* or *Magoh* in mice results in microcephaly (Mao et al., 2016). To understand if lower levels of EJC would lead to RS in the brain, we analyzed RNA-seq data from *Magoh* haploinsufficient mouse brain (*Magoh*^Mos2/+^). Highly included RS-exons in wild-type brain (PSI > 90%) showed increased skipping in the brain of *Magoh* haploinsufficient mice (Figure 6A), but not in control mice (Figure 6B). RT-PCR analysis validated 4 of these RS-exon skipping events in neuronal precursor cells (NPCs) derived from mouse embryonic stem cells (mESCs) that were transduced with shRNAs targeting *Eif4a3*, *Magoh*, or *Rbm8a* genes (Figures S6A–S6C). Importantly, two of the validated genes have previously been associated with microcephaly (Faheem et al., 2015). Since EJC happloinsuficiency primarily manifests a developmental brain phenotype, we wondered if the greater sensitivity of the brain tissue may reflect variable abundance of EJC components between tissues. Indeed, the expression level of *EIF4A3* and *MAGOH* mRNAs, as determined from genotype tissue expression (GTEx) data, is much lower in brain and muscle than in blood, lung, and breast, which suggests that a variation in functional EJC assembly might lead to physiological variations in the AS of RS-exons across tissues (Figure S6D).

### RS Leads to Physiological AS of RS-Exons

To gain insight into the role that RS plays in the AS of RS-exons, we returned to a more detailed analysis of PRPF8 crosslinking. In control cells, PRPF8 iCLIP revealed a crosslinking peak 12–14 nt upstream of exon-exon junctions formed by RS-exons, which was significantly higher when compared to non RS-exons (Figure 3B; independent samples t-test, p = 0.0016, t = 3.167). This indicates that some RS-exons can assemble spliceosome on the RS-5ss in the presence of EJC under physiological conditions. This is illustrated in the *MRPS5* gene, where a sharp peak of PRPF8 crosslinks is apparent upstream of the RS-exon both in control and eIF4A3 KD conditions and is accompanied by RS-exon skipping in RNA-seq (Figure 6C).

As an alternative approach to directly monitor RS, we examined the lariat-spanning reads that are present in total RNA-seq data by following previously established methods (Pineda and Bradley, 2018). Previous studies have focused on exon-inclusion lariats, where the sequence at the start of the intron is connected to the sequence upstream of the branchpoint (BP). Here, we searched for recursive-splicing lariats (RS-lariats), which arise from introns that link the 5′ end of an exon to the BP (Figure 6D). We identified 287 high-confidence pairings between the BP and the start of a preceding exon (putative RS-lariats; Table S6). We required reads supporting these high-confidence pairings to have a single mismatch lying at the inferred BP location, a stringent criterion that we previously found to be essential for accurate BP inference (Pineda and Bradley, 2018). These putative RS-lariats exhibited >4-fold enrichment for the –GU dinucleotide at the start of the exons containing such lariats, indicating that many of them likely reflect RS events. Approximately 44% of RS-lariats had adenine BPs, consistent with our previous observation that BPs corresponding to AS exhibited a decreased adenine frequency relative to those corresponding to constitutive splicing (Pineda and Bradley, 2018). In the RS-exon of *AP1G2* gene, we experimentally confirmed the RS event inferred by the computational analysis (Figures 6D and S6E). Taken together, PRPF8 iCLIP and analysis of splicing lariats indicate that RS can represent a mechanism for AS under physiological conditions.

### Inclusion of RS-Exons Increased Across Evolution

**Figure 7:**
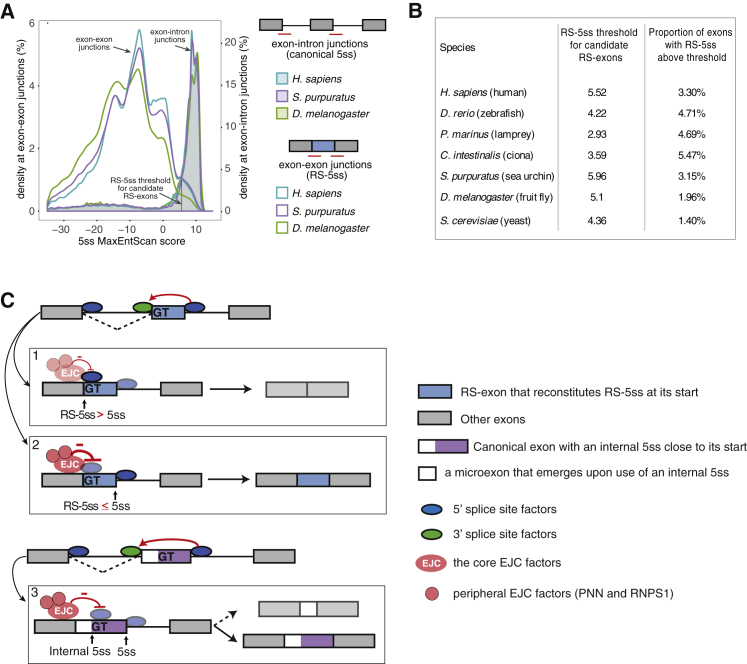
Analysis of RS-Exon Inclusion across Evolution (A) Density plot showing 5ss score distribution at exon-intron junctions (canonical 5ss, shaded lines) or at exon-exon junctions (RS-5ss, unshaded lines) for all internal annotated exons in human (blue), sea urchin (purple), or fruit fly (green). The red line represents the 5ss score is lower than 90% of exon-intron junctions in each species. This value is used as the RS-5ss threshold to quantify the proportion of putative RS-exons across species in (B). (B) A RS-5ss threshold was calculated as explained in (A) for 7 different species. Putative RS-exons for each species were calculated as the proportion of annotated exons that reconstitute a RS-5ss above the threshold. (C) Model for EJC-dependent repression of recursive splicing and microexon inclusion. Top panel: annotated putative RS-exons (in blue) are defined and spliced to their preceding exon to reconstitute a 5ss (RS-5ss) at the exon-exon junction, which leads to EJC deposition. Two outcomes can result from the second step of splicing: (1) the EJC does not efficiently repress the RS-5ss, either because it is a very strong RS-5ss or EJC assembly is deficient, and as a result, the RS-exon is recursively spliced; or (2) the EJC efficiently represses recursive splicing at most RS-5ss that are present in human mRNAs, most often leading to constitutive inclusion of RS-exons. Bottom panel: canonical exons that contain an internal 5ss close to their beginning (in white and purple) are defined and spliced to their preceding exon. (3) This splicing event leads to EJC deposition, which normally blocks the internal 5ss recognition, leading to an isoform that includes the full exon (bottom). However, if EJC does not repress the internal 5ss efficiently, its recognition leads to inclusion of a microexon (in white), while the rest of the exon (in purple) is removed in the second splicing step.

Finally, we wished to assess the potential for RS mechanism in regulating putative RS-exons across evolutionarily divergent species. To adjust the threshold for RS-5ss score to each species, we analyzed the distribution of canonical 5ss scores at exon-intron junctions of all internal exons annotated in 7 different species. We defined the 5ss score that includes 90% of all canonical 5ss in each species and then classified putative RS-exons that reconstitute a RS-5ss above this score (Figures 7A and 7B). All examined species from the deuterostome clade, from sea urchin to human, have a similar proportion of annotated RS-exons, whereas the proportion decreases approximately 2-fold in *Drosophila* and yeast (Figure 7B). The lower number of annotated RS-exons contrasts the large number of efficient intronic RS sites, which are more common in *Drosophila* (n = 197) than humans (n = 10) (Duff et al., 2015, Sibley et al., 2015). To understand if RS sites in *Drosophila* might be linked to cryptic RS-exons instead of annotated RS-exons, we examined previously defined *Drosophila* RS sites (Duff et al., 2015) to find that most of them are followed by an additional 5ss less than 100 nt downstream of the RS site (Figure S7A). These downstream 5ss tend to be more conserved than other 5ss-like sequences in the same introns (Figure S7A), which indicates that they contribute to the definition of cryptic RS-exons in *Drosophila* (Joseph et al., 2018), similar to the mechanism of RS in vertebrates (Sibley et al., 2015). To determine if RS of annotated RS-exons is repressed by the EJC in *Drosophila*, we performed RNA-seq in S2 cells treated with double-stranded RNAs (dsRNAs) against *mago*, *eIF4AIII*, and *LacZ* as control. Contrary to human cells and mouse brain respectively, we could not observe more skipping of exons with high RS-5ss score upon EJC factor depletion (Figure S7B). Thus, *Drosophila* contains a low number of ‘annotated RS-exons’ and we could not find any evidence of their sensitivity to EJC perturbation. This indicates that EJC assembly might not play an important role in the repression of RS in *Drosophila*, which could explain the large number of cryptic RS-exons in *Drosophila*.

## Discussion

In this study, we find that the human transcriptome contains hundreds of constitutive and alternative exons with RS-exon features. This observation can be explained by the fact that GT is the most common dinucleotide at exons starts (Sibley et al., 2016) and that the ends of exons are enriched for nucleotides compatible with 5ss. Once these “putative” RS-exons have been spliced to the preceding exon, they reconstitute a RS-5ss and thus gain the potential to be recursively spliced. However, splicing-dependent deposition of EJC efficiently represses RS of RS-exons, thus ensuring their efficient inclusion into mRNAs. Notably, all core EJC factors and the peripheral factors PNN and RNPS1 are required for this repressive function of the EJC. Conversely, stable tethering of assembly-deficient eIF4A3 alone is also sufficient for repression, which indicates that assembly of the whole EJC complex is required for its stable binding to RNA. We used PRPF8 iCLIP to confirm that upon EJC KD, spliceosome assembles at hundreds of RS-5ss that are formed by RS-exons. In addition to blocking RS-5ss, EJC also blocks other nearby 5ss, which leads to repression of cryptic microexons, which are derived from the beginning of annotated exons. Analysis of EJC haploinsufficient mouse brain and putative RS-exons across divergent species indicates that the function of the EJC in the repression of RS-exons is likely to be broadly conserved across deuterostomes.

### Mechanistic Insights into the EJC-Dependent Repression of RS

Since EJC deposition is not sequence dependent, it has been challenging to explain how it could regulate specific mRNAs. We now reveal a mechanism that provides insight into how the exonic sequences define the splicing functions of the EJC (i.e., its role in repressing the widespread presence of RS-5ss that are formed at junctions of specific exons in over a thousand mRNAs). In contrast to known splicing regulators, which promote inclusion of weak exons by increasing their definition, the EJC does not regulate definition or initial splicing of RS-exons. In fact, 5ss and 3ss scores of RS-exons are equal to or higher than those of non RS constitutive exons (data not shown). Instead, the EJC acts as a negative splicing factor by blocking spliceosome assembly at the RS-5ss reconstituted at the exon-exon junctions upstream of RS-exons. By repressing hundreds of RS-5ss, the EJC plays a major role in preserving transcriptome integrity in humans. Due to the large number of RS-5ss in the transcriptome and their positioning next to the EJC, RS-5ss are likely to be the primary target for EJC-dependent repression, but as evidenced by the new class of EJC-repressed microexons in our study and a parallel study (Boehm et al., 2018), other types of cryptic splice sites can also be repressed by the EJC in part-spliced transcripts.

The EJC is the primary RNP deposited on spliced mRNAs, but most functional studies of the EJC have focused on its cytosolic role in premature termination codon recognition during NMD (Le Hir et al., 2016). The role of the EJC in repressing hundreds of RS events has opened a new opportunity to examine its nuclear function from the moment the EJC starts assembling on the partly spliced RNA (Figure 7). By using assembly-deficient mutants of eIF4A3 and MAGOH, we found that these proteins cannot individually bind to pre-mRNA to repress RS; instead, assembly of the EJC, including the peripheral components PNN and RNPS1, is required for the repressive capacity. However, stable tethering of an assembly-deficient eIF4A3 mutant (401/402) is sufficient to repress RS. This confirms the previous findings that endogenous eIF4A3 needs to assemble the whole EJC in order to achieve stable binding (Gehring et al., 2009). If stable binding is achieved artificially via the MS2 system, then it can repress RS independently of the EJC.

The second mechanistic question addresses why specific RS-exons are more prone to RS than others. We found that EJC-sensitive RS-exons have a preferential splicing order compared to other exons, with an increased propensity to have their upstream intron spliced first. This is consistent with the trend for slightly stronger 3ss at EJC-sensitive RS-exons compared to non sensitive exons, which could increase the splicing kinetics of the preceding intron, and slightly weaker 5ss, which could decrease the splicing kinetics of the following intron. This preferential splicing order is also consistent with the expectation that RS is only possible if the upstream intron is spliced first to create the RS-5ss that is then used for the second splicing step. If the repressive capacity of the EJC may not sufficient to repress the RS-5ss, then the RS-exon is alternatively spliced or fully skipped through RS (Figure 7C, 1). However, most human putative RS-exons are constitutively included, and many are dependent on the EJC for their inclusion (Figure 7C, 2). A third possibility involves the presence of an internal 5ss close to the beginning of exons, which is within the range of EJC repression; upon perturbation of the EJC, recognition of the internal 5ss leads to inclusion of a cryptic microexon (Figure 7C, 3). These appear to be mechanistically distinct from the previously studied microexons, which are defined before the first step of splicing by proteins such as nSR100 (Irimia et al., 2014). Inclusion of cryptic microexons is regulated at the level of the part-spliced transcript, where absence of the EJC causes the splicing machinery to reposition from the canonical to the internal 5ss of the host exon.

### The Function of RS Regulation during Development

While it is clear that the regulation of exon definition is responsible for most AS, we now demonstrate that RS also contributes to the physiological regulation of AS. PRPF8 iCLIP reveals spliceosomal recognition of RS-5ss formed by specific alternative RS-exons in unperturbed cells, without any depletion of EJC components. Moreover, we provide further evidence in support of endogenous RS by analysis of lariat-spanning reads from RNA-seq and their validation with PCR. Analysis of RNA-seq data from *Magoh* haploinsufficient mouse brain also shows that the role of the EJC in repressing RS-exon skipping is conserved at least from mouse to humans, if not more broadly. The analysis of genes affected by RS-exon skipping events in the brain might also provide a clue into the microcephaly phenotype of this mouse model. Genes that contain abnormally skipped RS-exons in *Magoh* haploinsufficient brain include *Aspm* and *Cenpj*, two genes previously associated with autosomal-recessive microcephaly that encode proteins essential for mitotic cell progression during embryonic neurogenesis (Faheem et al., 2015).

We identified hundreds of EJC-sensitive RS-exons in HeLa cells, and it is likely that the EJC plays a role in regulating many additional alternative RS-exons in various cell types. An important question for future research will be to understand if the capacity of the EJC to promote RS-exon inclusion might vary during development or between cell types, which would be plausible for several reasons. The expression level of EJC factors across different tissues from GTEx data suggests their protein abundance is likely to vary across cell types, and additionally, the assembly of EJC at individual junctions could be regulated in an exon-specific manner by factors such as SR proteins (Singh et al., 2012). Moreover, we identified two RS-exons that are more sensitive to depletion of RNPS1 and PNN than the core EJC factors. Such exons could be affected by differential activities of the peripheral factors, which could be regulated by post-translational modifications or mutually exclusive binding, such as the binding of PNN or ACIN1 to RNPS1 (Murachelli et al., 2012). It has also been shown that EJC binding can be affected by RNA secondary structures (Mishler et al., 2008, Singh et al., 2012). It remains to be seen if these variables might affect the efficiency of the EJC in blocking RS and if the release from EJC repression could serve as a mechanism for regulating the physiological AS of specific RS-exons.

### Evolutionary Implications of the EJC-Dependent RS Repression

Our results indicate that the efficiency of EJC-dependent repression of RS might have increased in the evolution of deuterostomes, as evident by comparison of a perturbed EJC on RS-exons in *Drosophila*, mouse, and human. This is consistent with the increased proportion of RS-exons in the transcriptomes of deuterostomes. Our experiments with mutant splicing reporters demonstrate that a single point mutation in the RS-5ss can be sufficient to convert a cryptic RS-exon into an alternative or constitutive exon. While such mutations could contribute to the evolution of EJC-regulated RS-exons, they could also lead to disease by activating inclusion of aberrant cryptic exons. We also found that mutations that increase the strength of RS-5ss can increase the skipping of canonical exons, which should be taken into account when interpreting the mechanisms of disease-causing mutations that are located near the start of exons.

## STAR★Methods

### Key Resources Table

**Table:** 

REAGENT or RESOURCE	SOURCE	IDENTIFIER
**Antibodies**		

Rabbit anti-PRPF8 Antibody	Bethyl	Cat#A303-922A; RRID: AB_2620271
Rabbit anti-eIF4AIII Antibody	Abcam	Cat#ab32485; RRID: AB_732124
Mouse anti-RBM8A Antibody	SCBT	Cat#sc-32312; RRID: AB_2178827
Rabbit anti-MAGOH Antibody	Abcam	Cat#ab180505
Rabbit anti-UPF1 Antibody	Abcam	Cat#ab109363; RRID: AB_10861979
Mouse anti-FLAG Antibody	Sigma	Cat#F1804: RRID: AB_262044
Mouse anti-GFP Antibody	Santa Cruz	Cat#sc-9996; RRID: AB_627695
Rabbit anti-GAPDH Antibody	Cell signaling	Cat#2118L; RRID: AB_561053
Mouse anti-GAPDH Antibody	Abcam	Cat#ab8245; RRID: AB_2107448

**Bacterial and Virus Strains**		

NEB 5-alpha Competent *E. coli* (High Efficiency)	New England Biolabs	Cat#C2987I

**Biological Samples**		

Human Brain, Cerebellum Total RNA	Takara Bio	Cat#636535

**Chemicals, Peptides, and Recombinant Proteins**		

TRIzol Reagent	Thermo Fisher Scientific	Cat#15596026
Phusion High-Fidelity DNA Polymerase (2 U/μL)	Thermo Fisher Scientific	Cat#F530L
10 mM dNTP Mix	Thermo Fisher Scientific	Cat#18427088
UltraPure Agarose	Thermo Fisher Scientific	Cat#16500500
BlueJuice Gel Loading Buffer	Thermo Fisher Scientific	Cat#10816015
Kanamycin Sulfate	Thermo Fisher Scientific	Cat#BP906-5
Zeocin Selection Reagent	Thermo Fisher Scientific	Cat#R25001
ESGRO Recombinant Mouse LIF Protein	Millipore	Cat#ESG1107
PD 0325901	Sigma	Cat#PZ0162-25MG
CHIR99021	Sigma	Cat#SML1046-25MG
Recombinant Mouse FGF basic Protein	R&D Systems	Cat#3139-FB-025
Lipofectamine RNAiMAX Transfection Reagent-1.5 mL	Thermo Fisher Scientific	Cat#13778150
Lipofectamine 2000 Transfection Reagent-1.5 mL	Thermo Fisher Scientific	Cat#11668019
Endoporter	GeneTools	http://www.gene-tools.com/endo_porter
AMPure XP, 5 mL	Agencourt	Cat#A63880
RIPA Buffer	Sigma-Aldrich	Cat#R0278-50ML
cOmplete(TM) Protease Inhibitor Cocktail	Sigma-Aldrich	Cat#11697498001
NuPAGE Novex 4-12% Bis-Tris Protein Gels, 1.0 mm, 10 well	Thermo Fisher Scientific	Cat#NP0321BOX
Lipofectamine 3000 Transfection Reagent-1.5 mL	Thermo Fisher Scientific	Cat#L3000015
Lenti-X Concentrator, 100 mL	Takara Clontech	Cat#631231
Puromycin	Takara Clontech	Cat#631305
Blasticidin S HCl	ThermoFisher Scientific	Cat#R21001
Hygromycin B (50mg/ml)	ThermoFisher Scientific	Cat#10687010
Doxycycline hyclate	Sigma-Aldrich	Cat#D9891
Novex TBE Gels, 6%, 10 well-1 box	Thermo Fisher Scientific	Cat#EC6265BOX

**Critical Commercial Assays**		

DNA Clean & Concentrator-5	Zymo Research	Cat#D4014
Zymoclean Gel DNA Recovery	Zymo Research	Cat#D4007
SuperScript IV First-strand synthesis system	Thermo Fisher Scientific	Cat#18091050
Zero Blunt TOPO PCR Cloning Kit for Sequencing, with One Shot TOP10 Chemically competent E.coli	Thermo Fisher Scientific	Cat#K2875J10
SENSE mRNA-Seq Library Prep Kit v2 for HiSeq, 96 preps	Lexogen	N/A
QIAxcel DNA Screening Kit (2400)	QIAGEN	Cat#929004
Fast SYBR Green Master Mix	Thermo Fisher Scientific	Cat#4385612
ImmoMix	BIOLINE	Cat#BIO-25020
Maxwell RSC simplyRNA Cells Kit	Promega	Cat#AS1390
High-Capacity cDNA Reverse Transcription Kit-200 reactions	Thermo Fisher Scientific	Cat#4368814
MEGAclear Transcription Clean-Up Kit	Thermo Fisher Scientific	Cat#AM1908
MEGAscript T7 Transcription Kit	Thermo Fisher Scientific	Cat#AM1333

**Deposited Data**		

Original gel and capillary electrophoresis images and quantification for all figures	This study	https://doi.org/10.17632/7f9z9yxcx8.1
PRPF8 iCLIP after eIF4A3 knockdown in HeLa cells	This study	Raw data accessible via https://www.ebi.ac.uk/arrayexpress/ E-MTAB-7269 and both raw and processed data available at https://imaps.genialis.com
mRNA-seq after EJC component knockdown in S2 cells	This study	E-MTAB-7271. Accessible via https://www.ebi.ac.uk/arrayexpress/
RNA-seq data from ENCODE	https://www.encodeproject.org/search/?type=Experiment&assay_title=shRNA+RNA-seq&limit=all	Table S7
RNA-seq data from K562 cells enriched for lariats	(Mercer et al., 2015)	GEO: GSE53328
RNA-seq data from K562 and NALM-6 cells enriched for lariats	(Taggart et al., 2017)	SRA: SRP094107
RNA-seq data from HeLa cells after EJC KD	N/A	GEO: GSE63091
RNA-seq data after PNN KD in corneal epithelial cells	N/A	GEO: GSE73060
RNA-seq data after Acinus KD in HeLa cells	N/A	GEO: GSE81460
RNA-seq data after KD of NMD factors in HeLa cells	N/A	GEO: GSE86148
RNA-seq data after KD of EJC auxiliary factors in human lymphoblastoid cell lines	N/A	GEO: GSE52834
RNA-seq data after KD of SR proteins in mouse P19 cells	N/A	GEO: GSE69733
RNA-seq data of EJC haploinsufficient mouse neocortices	N/A	GEO: GSE85576
eIF4A3 and BTZ iCLIP data	(Hauer et al., 2016)	ArrayExpress: E-MTAB-4215.
RNA-seq HeLa control samples	NCBI sequence read archive	SRA: SRR514854
RNA-seq HeLa control samples	NCBI sequence read archive	SRA: SRR514855
*Homo sapiens* reference genome NCBI build 37, GRCh37	Genome Reference Consortium	https://www.ncbi.nlm.nih.gov/projects/genome/assembly/grc/human/
*Danio rerio* reference genome (GRCz10 Build 88)	Genome Reference Consortium	N/A
*Drosophila melanogaster* reference genome (BDGP6 Build 88/ BDGP R5/dm3)	Berkeley Drosophila Genome Project	http://www.fruitfly.org
*Schizosaccharomyces pombe* reference genome (ASM294v2 Build 35)	Ensembl	https://fungi.ensembl.org/Schizosaccharomyces_pombe/Info/Index
*Ciona intestinalis* reference genome (KH)	Ensembl	http://www.ensembl.org/Ciona_intestinalis/Info/Index
*Petromyzon marinus* reference genome (7.0)	Ensembl	http://www.ensembl.org/Petromyzon_marinus/Info/Index
*Strongylocentrotus purpuratus* reference genome (3.1)	Ensembl	https://metazoa.ensembl.org/Strongylocentrotus_purpuratus/Info/Annotation/

**Experimental Models: Cell Lines**		

Human: K562 cells	ATCC	ATCC number CCL-243
Human: HeLa Flp-In T-Rex cells	N/A	Prof. Stephen Taylor (University of Manchester)
Human: HEK293T cells	European Collection of Authenticated Cell Cultures (ECACC)	12022001
Human: HEK293 cells	ATCC	ATCC number CRL-1573
Mouse: 46C cells	(Stavridis and Smith, 2003)	N/A
Drosophila: S2 cells	Drosphila Genomics Resource Center (DGRC)	S2-DGRC

**Oligonucleotides**		

Stealth RNAi siRNA Negative Control, Med GC	Thermo Fisher Scientific	Cat#12935300
KPNA1 Morpholino	GeneTools	5′ GAATATCATCCCCTGTGACAATGTT 3′
Control Morpholino	GeneTools	5′ CCTCTTACCTCAGTTACAATTTATA 3′
EIF4A3 Stealth siRNA	Thermo Fisher Scientific	Cat#HSS114709
UPF1 Stealth siRNA	Thermo Fisher Scientific	Cat#HSS109172

**Recombinant DNA**		

QX DNA Size Marker 50–800 bp (50ul)	QIAGEN	Cat#929561
pcDNA 3.1(+) Mammalian Expression Vector	Thermo Fisher Scientific	Cat#V79020
pOG44 Flp-Recombinase Expression Vector	Thermo Fisher Scientific	Cat#V600520
pcDNA5 FRT/TO Vector Kit	Thermo Fisher Scientific	Cat#V652020
pLKO.1 puro	N/A	Addgene plasmid # 8453
pCMV-VSV-G	N/A	Addgene plasmid # 8454
pMDLg/pRRE	N/A	Addgene plasmid # 12251
pRSV-Rev	N/A	Addgene plasmid # 12253
pCI-neo FLAG eIF4A3	(Gehring et al., 2009)	N/A
pCI-neo FLAG MAGOH	(Gehring et al., 2009)	N/A
pCI-neo FLAG eIF4A3 E188R	(Gehring et al., 2009)	N/A
pCI-neo FLAG eIF4A3 401/402	(Gehring et al., 2009)	N/A
pCI-neo FLAG MAGOH E20R	(Gehring et al., 2009)	N/A
pCI-neo FLAG eIF4A3 E188Q	This study	Oligos for cloning detailed in Table S2
pMS2-GFP	This study	Oligos for cloning detailed in Table S2
pMS2-eIF4A3	(Gehring et al., 2009)	N/A
pMS2-eIF4AIII 401/402	(Gehring et al., 2009)	N/A
pcDNA3 and pcDNA5 splicing reporters	This study	Sequences detailed in Table S5

**Software and Algorithms**		

Bowtie2	N/A	http://bowtie-bio.sourceforge.net/bowtie2/index.shtml
R/Bioconductor	N/A	https://www.bioconductor.org/
R/dplyr	N/A	http://cran.r-project.org/web/packages/dplyr/index.htmld
R/ggplot2	N/A	https://cran.r-project.org/web/packages/ggplot2/
STAR RNA aligner	N/A	https://github.com/alexdobin/STAR
Samtools version 1.3.1	N/A	http://samtools.sourceforge.net/
Bedtools (v.2.17.0)	N/A	https://academic.oup.com/bioinformatics/article-lookup/doi/10.1093/bioinformatics/btq033
MAxEntScan	(Yeo and Burge, 2004)	http://genes.mit.edu/burgelab/maxent/Xmaxentscan_scoreseq.html
MAJIQ: Modeling Alternative Junction Inclusion Quantification	N/A	https://majiq.biociphers.org/
RSEM and EBSEQ	N/A	https://github.com/deweylab/RSEM
R	R Project for Statistical Computing	RRID: SCR_001905; http://www.r-project.org/
RNAfold	N/A	http://rna.tbi.univie.ac.at/cgi-bin/RNAWebSuite/RNAfold.cgi
iMaps webserver	N/A	https://imaps.genialis.com/
Hisat2 version 2.0.5	N/A	https://ccb.jhu.edu/software/hisat2/index.shtml
Cutadapt	N/A	https://cutadapt.readthedocs.io/en/stable/guide.html

### Contact for Reagent and Resource Sharing

Further information and requests for resources and reagents should be directed to and will be fulfilled by the Lead Contact, Jernej Ule (jernej.ule@crick.ac.uk).

### Experimental Model and Subject Details

HeLa, HeLa Flp-In T-Rex, HEK293 and HEK293T cells (all of female origin) were cultured in Dulbecco’s modified Eagle medium. K562 cell (female origin) was cultured in Iscove’s Modified Dulbecco’s Medium. All cells were supplemented with 10% fetal bovine serum, grown at 37°C with 5% CO2 injection, and routinely passaged twice a week. HeLa Flp-In T-Rex cells were selected in the presence of 3ug ml^-1^ of blasticidin and 50ug ml^-1^ of Zeocin. Mouse ESCs (46C) cells (Ying et al., 2003) were grown feeder free on 0.2% gelatinized cell culture plates in 2iL media (2i+LIF) (Stavridis and Smith, 2003). S2 cells were grown at room temperature in *Drosophila* Schenider’s medium containing 10% fetal bovine serum. Cell lines were confirmed to be mycoplasma-free with repeated testing, using the MycoAlert mycoplasma detection kit (Lonza). Cells were not authenticated by us, but retrieved from trusted sources as listed in the Key Resources Table.

### Method Details

#### Experimental methods

##### Plasmids

*Splicing reporters.* Main and alternative *CADM2* (m*CADM2*/a*CADM2*) splicing reporters had been previously described (Sibley et al., 2015). To generate *KPNA1* splicing reporter, RS-exon and both upstream and downstream exons were cloned in HindIII-EcoRI sites of pcDNA3 plasmid. Each exon was flanked by the nearest 100 nucleotides of their respective introns. Part-spliced constructs were generated by removing the intronic sequence between the RS-exon and its preceding exon. Sequences were obtained by gene synthesis (GeneArt, Life Technologies). If necessary, splicing reporters were sub-cloned from pcDNA3 to pcDNA5/FRT/TO (pcDNA5) plasmid using HindIII-NotI restrictions sites. In *PSMA3-AS1* splicing reporter, RS-exon and both upstream and downstream exons flanked by 100 nucleotides of intronic sequence were cloned in pcDNA5 HindIII-NotI restriction sites by cross-over PCR using oligonucleotides detailed in Table S2. To generate *RPS2* splicing reporter, non RS-exon and both upstream and downstream exons flanked by their respective introns were cloned in HindIII-NotI sites of pcDNA5 plasmid. Sequences were obtained by gene synthesis (GeneArt, Life Technologies). All mutant splicing reporters with the sequences provided in Figure 3G were obtained by gene synthesis (in *KPNA1*, a*CADM2 and RPS2*) or by site-directed mutagenesis using oligonucleotides detailed in Table S2 following Quick Change Site-directed mutagenesis instructions (Agilent Technologies). To generate splicing reporters with Stem Loop 2 (SL2) sequence in the upstream exon, nucleotides in positions −9 to −34 relative to the exon junction were replaced by AAAGCACGAGCATCAGCCGTGCCTC sequence. Wild-type SL2 sequences were obtained by gene synthesis in *KPNA1* or cross-over PCR in m*CADM2* splicing reporters respectively. To generate SL2 mutant reporters, C nucleotide in −20 position was mutated to a G, as this position must be a pyrimidine for tight binding to MS2 coat protein (Valegârd et al., 1997). SL2 mutant plasmids were created by site-directed mutagenesis using oligonucleotides detailed in Table S2. To move SL2 sequences further upstream the exon-exon junction, sequences were obtained by gene synthesis and cloned into *mCADM2* Unspl SL2 Wt splicing reporter using HindIII and AscI restriction sites. Microexon containing *KPNA1* Unspliced and Part-spliced wild-type and mutant sequences were obtained by gene synthesis and cloned into pcDNA5/FRT/TO (pcDNA5) plasmid using HindIII-NotI restrictions sites. Sequences of all splicing reporters are detailed in Table S5. Rescue plasmids: pCI-neo FLAG eIF4A3 E188Q construct was generated by site-directed mutagenesis. pMS2-GFP plasmid was generated by PCR amplification of GFP ORF in pGFP plasmid and subsequent cloning into pMS2-eIF4A3 using XhoI and NotI restriction sites to replace eIF4A3 with GFP. The oligonucleotides used for cloning are detailed Table S2.

*Cell lines.* To generate stable HeLa Flp-In T-Rex cell lines, splicing reporters cloned in pcDNA5 plasmids were transiently co-transfected with pOG44 plasmid following manufacturer’s instructions. Positive selection of clones was done with 100-250 ug ml^-1^ of hygromycin B. To induce expression of the stably integrated splicing reporter, media was supplemented with 200ng ml^-1^ of doxycycline. pcDNA5 *KPNA1* Unspliced Mut plasmid was used to generate *KPNA1* RS-5ss Mutant cell line. pcDNA5 *aCADM2* Unspliced Wt and Mut plasmids were used to produce *aCADM2* wild-type or RS-5ss mutant cell lines respectively. pcDNA5 *PSMA3-AS1* Wt and Mut plasmids were used to create *PSMA3-AS1* wild-type and RS-5ss mutant cell lines respectively. Mouse ESCs (46C) were grown feeder free on 0.2% gelatinized cell culture plates in 2iL media (2i+LIF) (Stavridis and Smith, 2003). To differentiate mESCs into neural precursor cells (NPCs), single cells were plated at a density of 10,000 cells cm^-2^ in N2B27 media supplemented with 10ng ml^-1^ of bFGF (R&D) for 3 days and then cultured in N2B27 media without growth factors (Gouti et al., 2014).

*siRNAs and Oligonucleotides.* siRNA delivery was performed using Lipofectamine RNAiMax according to manufacturer’s recommendations. Final concentration of siRNAs was 20nM. The sequences/reference numbers of siRNAs used are listed in Table S1 or Key Resources Table. Cells were collected for analysis 48 hours after initial siRNA transfection. MAGOH KD was achieved by combining siRNAs that target MAGOH and MAGOHB paralog genes. eIF4AIII, RBM8A, MAGOH and UPF1 KDs were done in HeLa *aCADM2* Flp-In to examine *aCADM2* splicing pattern. eIF4AIII, PNN, RNPS1, SUPV3L1 and ASSC1 KDs were performed in HeLa Flp-In T-REx host cell line. Morpholinos (GeneTools, USA) were delivered at 10uM final concentration using Endoporter reagent in HeLa Flp-In T-REx *KPNA1* Mut cell line or mouse NPCs. *KPNA1* morpholino was reverse complement to nucleotides −9 to −34 in the exon preceding the RS-exon, relative to the exon junction. The sequence was determined based on the local RNA structure (as determined by RNAfold) as an accessible single-stranded region.

*Transient transfection.* All transient transfections were carried out using Lipofectamine 2000 in HeLa or HEK293 cells grown at ≈80% confluency in six-well plates. For unspliced and part-spliced splicing reporter experiments, 1ug of pcDNA3 *KPNA1* or *aCADM2* unspliced, part-spliced or part-spliced mutant plasmids was transfected in HeLa Flp-In T-Rex host cell line and cells were harvested 24 hours after transfection. For KD and rescue experiments, 20nM siRNAs and 1.5ug of pGFP or pCI-neo plasmids were transfected in HeLa *aCADM2* wild-type cell line. For tethering rescue experiments, 500ng of pcDNA5-SL2 splicing reporters were co-transfected with a pGFP or pMS2 expression reporters in HEK293 cell lines. In both rescue experiments cells were harvested 48 hours after transfection for RNA and protein isolation.

*dsRNA production and treatment.* dsRNA production and treatment was done following the procedure previously described in Aerne et al. (2015). Briefly, dsRNAs against *mago* and *eIF4AIII* mRNAs were synthesized by *in vitro* transcription using T7 MEGAscript and MEGAclear kits. PCR products used as templates for *in vitro* transcription were obtained by PCR using *Drosophila* genomic DNA as template and the primers listed in Table S3. For knockdown, one million S2 cells were plated per 6 well in 2 m of serum containing medium. After 2-3h, 25ug of dsRNA were dissolved in 1ml of serum free medium, which was added to the cells. Cells were soaked for 30 minutes, after that 2ml of additional serum containing medium was added. Cells were lysed 3 days later and RNA-seq libraries were generated using SENSE mRNA-Seq Library Prep kit V2, following manufacturer’s instructions.

*RNA extraction and cDNA analysis.* RNA was extracted from cell pellets using Maxwell RSC simply RNA cells kit in Maxwell RSC instrument following manufacturer’s instructions. RNA was reverse transcribed using High-capacity cDNA synthesis kit using random primers and standard protocol. A total of 1 μg was used in each reaction and cDNA then diluted 1:10. For RT-PCR, 2 μL of diluted sample was used for each subsequent PCR reaction using 2X Immomix Master Mix and each primer at a final concentration of 0.5μM. PCR products were visualized in a QIAxcel Advanced System using a QIAxcel DNA Screening kit (QIAGEN) following manufacturer’s instructions. Splicing pattern of endogenous RS-exons was monitored using oligonucleotides detailed in Table S3. Splicing pattern of transiently or stably expressed splicing reporters was analyzed using a Forward oligo specific for the gene under study and BGHpA Rv oligo. For qPCR, 5 μL of diluted sample was used for each reaction using SYBR green PCR mastermix and each primer at a final concentration of 0.2μM in a QuantStudio 6 Flex Real-Time PCR System. Oligos used for qPCR are listed in Table S4.

*PRPF8 iCLIP.* iCLIP data for PRPF8 was derived from HeLa cells treated with either control or eIF4A3 siRNA, as described above. Four replicate samples for each condition were generated. An antibody against endogenous PRPF8 was used. The iCLIP method followed was described in Huppertz et al. (2014), with the following modifications. For the first replicate of each condition, protein-RNA complexes were visualized using a preadenylated, infrared dye labeled L3 adaptor (Zarnegar et al., 2016) with the following sequence:

/5rApp/AG ATC GGA AGA GCG GTT CAG AAA AAA AAA AAA /iAzideN/AA AAA AAA AAA A/3Bio/

For the following three replicates of each condition, an unlabelled L3 adaptor was ligated to the RNA with a barcode:

/5rApp/WN *XXX* AGA TCG GAA GAG CGG TTC AG/3Bio/

This allowed these replicates to be multiplexed prior to SDS-PAGE. The transfer of protein-RNA complexes was adapted for the high molecular weight of PRPF8 by running an overnight transfer at 4C and at 15V with the addition of 0.05% SDS to the transfer buffer. Reverse transcription was performed with barcoded primers containing UMIs:

/5Phos/ WWW *XXXXX* NNNN AGATCGGAAGAGCGTCGTGAT /iSp18/ GGATCC /iSp18/ TACTGAACCGC

Purification of cDNAs following reverse transcription and circularization was performed using AMPure XP beads (Beckman-Coulter, USA) and isopropanol. Libraries were sequenced as single end 100bp reads on Illumina HiSeq 4000.

*Immunoblot analysis.* Cell pellets were lysed in RIPA buffer with protease inhibitors. 10-20μg of total protein extracts were resolved in Novex Tris-Glycine 4%–12% gels, transferred to nitrocellulose membranes and blotted against eIF4AIII, RBM8A, MAGOH, UPF1, FLAG or GFP proteins. GAPDH was used as a loading control. Category numbers for all antibodies are listed in Key Resources Table.

*Lentivirus production and infection.* shRNAs against *Eif4a3*, *Rbm8a* and *Magoh* genes were cloned into the AgeI/EcoRI sites of pLKO.1 puro plasmid using oligonucleotides detailed in Table S2. Each plasmid was co-transfected with pVSV-G, pRSV and pRev plasmids in 293T cells using Lipofectamine 3000 following manufacturer’s instructions. 24 and 48 hours after transfection cell media was collected and concentrated using Lenti-X Concentrator. Lentivirus particles were later used to infect neural precursor cells and selected with 2μg ml-1 of Puromycin for 48 hours. Cells were harvested for RNA and protein analysis.

*Direct lariat sequencing.* Direct lariat sequencing for recursive lariat in *AP1G2* gene was the same procedure described previously in (Pineda and Bradley, 2018). The primers used are listed in Table S3.

#### Computational Analyses

Customised data analysis pipelines were implemented using python (v.2.7.2), Bedtools (v.2.17.0) and R (v. 3.2.4).

##### Defining the internal exons

Only exons internal to protein-coding genes were studied as defined by the presence of RNA-seq reads spanning both the upstream and downstream junctions within each dataset (described below). Exon positions were determined by the following annotations: GENCODE version 19 annotation (human genome build hg19), GENCODE version M11 (mouse genome build mm10) and ENSEMBL version 86 (fruit fly genome build dm6).

##### RNA-seq processing pipeline

All raw sequence reads were aligned to human (hg19), mouse (mm10) or fruit fly (dm6) genomes respectively using STAR aligner v2.4 (https://github.com/alexdobin/STAR) with basic GENCODE annotations for canonical splice sites. Junction files were collected and collated from samples within the same study. For each internal, protein-coding exon, the up- and downstream splice junctions with highest read count were selected. The skip junction was then defined as the junction connecting the upstream exon to the downstream exon based on these junctions. Filtering was done to remove exons with insufficient total junction read counts across samples. Filtering criteria for each dataset is listed below:

• ENCODE: 200 total junction reads per exon
• Other public datasets (including GEO: GSE63091, GSE52834, GSE73060, GSE81460, GSE86148, GSE85576, GSE69733): 10 total junction reads per exon

Percent-spliced-in (PSI) was then calculated per sample as:

PSI=50∗(upstream+downstreamjunctions)/(skippingjunction+50∗(upstream+downstreamjunctions))

Change in exon inclusion (ΔPSI) for KD experiments was calculated based on difference between PSI in a specific KD and the mean PSI of control samples.

##### Defining non RS and RS exon categories

MaxEnt splice site scoring software was used to determine the score of 5′ splice sites (5ss) (Yeo and Burge, 2004). To determine the threshold for RS-5ss to identify candidate RS-exons, we first determined for each species the MaxEntScan score present at 90% of canonical 5ss for these species. For this analysis, we used all exon-intron junctions of annotated exons for the species where the MaxEntScan score of 5ss was > 0. This was done because the distribution of MaxEntScan scores across exon-intron junction was bimodal, with the major peak of 5ss was found between 0 and 12 (Figure 7A). We suspect that the minor peak of 5′ss with scores between −30 and 0 are more likely to be spurious sites that result from false annotation, or non-canonical splice sites that are present at a low proportion at true exons (Sibley et al., 2016). For human transcriptome, the threshold was 5.52, For each exon, the potential reconstituted 5′ splice site (hereafter: RS-5ss) was then examined in the same way after linking each exon to the preceding exon. For analysis of human exons, the end of the preceding exon was defined by RNA-seq, such that the upstream splice junction with most reads for that exon was used. For other species, only the annotated position of the nearest preceding exon was used, as defined by ENSEMBL annotations for the following species: *Schizosaccharomyces pombe* (ASM294v2), Danio rerio (GRCz10), *Drosophila melanogaster* (BDGP6), *Homo sapiens* (GRCh37.75), Ciona intestinalis (KH), Petromyzon marinus (Pmarinus_7.0) and Strongylocentrotus purpuratus (3.1). All exons with RS-5ss > threshold at the exon-exon junction were then considered as potentially recursive “RS exons,” while the remaining exons were considered “nonRS exons.”

##### Testing for differences in ΔPSI between groups of exons with similar RS-5ss score

For each experiment, ΔPSI values for each exon category (alternative (15% < PSI < 85%), highly-included alternative (85% < PSI < 98%) and constitutive exons (PSI > 98%) were calculated by comparing two groups of exons, one above and one blow a threshold defined by the RS-5ss score. The RS-5ss score threshold produced two groups of exons which could be tested for skew in ΔPSI values using the Wilcox rank sum test. A range of RS-5ss score threshold values was then examined, from −20 to 8, in order to determine how the ΔPSI values were affected by the threshold. The signed P value (based on the sign of the test statistic and the generated P value) for each RS-5ss score threshold are shown in Figures 1G, 2A, and 2B. For all RNA-seq samples showing significant signed P value for exons with RS-5ss > 4 (indicating that their inclusion decreased compared to other exons), the data were reanalysed using MAJIQ to recalculate PSI. ΔPSI estimates for all further analyses were calculated as the difference between the control sample PSI value and the knockdown sample PSI value.

##### Differential gene expression of SUPV3L1

RSEM transcript estimate data was downloaded from https://www.encodeproject.org/ for both SUPV3L1 (K562 cell line: ENCBS534VZR) and its Control (K562 cell line: ENCSR778SIU). EBSEQ was run using default settings to produce differential gene expression statistics.

##### Evolutionary comparison of 5′ splice sites

In order to estimate the prevalence of potential RS-exons across evolution, we developed a simple splice site scoring algorithm that took into account the characteristics of 5′ splice sites (5ss) present in each species. A collection of top 1000 most prevalent 9mers were collected from canonical 5ss. The presence of these the 1000 canonical 5ss 9mers was then examined at each potential RS-5ss by using ENSEMBL exon annotations for the following species; *Schizosaccharomyces pombe* (ASM294v2 Build 35), Danio rerio (GRCz10 Build 88), *Drosophila melanogaster* (BDGP6 Build 88), *Homo sapiens* (GRCh37.p13 Build 88).

##### iCLIP data analysis and visualization

Mapping of the eIF4A3 and BTZ iCLIP data (Hauer et al., 2016) to the UCSC hg19/GRCh37 genome assembly was performed using STAR (version 020201) alignment software with default settings. cDNAs with the same unique molecular identifier (UMI) that mapped to the same genomic position were considered as PCR duplicates and collapsed to a single cDNA. All normalizations were performed in R (version 3.1.0) together with the “ggplot2” and the “smoother” packages for visualizing the results. To visualize the normalized cDNA coverage of eIF4A3 and BTZ iCLIP data (Hauer et al., 2016), we used the full sequence of cDNAs, since these cDNAs represent crosslink sites at their starts, but also inform on RNase protection by EJC over their whole sequence (Haberman et al., 2017). Each density graph shows normalized cDNA coverage around the 5ss upstream to EJC-sensitive and insensitive RS-exons. For each group of exons, we measured the cDNA coverage from RNA-seq data and calculated the normalization factor f(xi) in the following way:

f(xi)=sum(xi)/(sum(xi)+sum(xi+1)…+sum(xn))

where f is the normalization factor and x is the cDNA coverage.

cDNA coverage was then normalized by the normalization factor f(xi) for each exon group.

Processing of the PRPF8 iCLIP data was performed using the iMaps webserver (https://imaps.genialis.com/). The data was mapped to the GRCh38/GENCODE v27 genome assembly using STAR (version 2.6.0) with default settings. PCR duplicates and reads that did not map uniquely to the genome were discarded. Reads for each replicate were separated based on the presence or absence of a junction within the read. For junction-containing reads, only reads that start and end in an annotated exon were considered. Junction-containing reads were then classified to RS or non-RS groups on the basis of the read ending in an RS or non-RS exon, respectively. For each read, the distance from the read start to the end of the exon was calculated. The frequency of read start-distance for each group was plotted as a frequency polygon using R (version 3.5.0) and and the ggplot2 package. The LOESS algorithm was used to smooth the data with span set to 0.2. The data was normalized on the basis of the total number of reads for each sample’s library and the total number of exons in the exon class surveyed for that group (e.g., RS versus non-RS). Reads ending in exons that contained only a single read across all samples were excluded from the analysis. EJC-sensitive exons were defined as those that showed a greater than 0.05 dPSI between eIF4A3 knockdown and control in the ENCODE RNaseq data. Junction-absent reads were classified in to RS or non-RS groups on the basis of the RS status of the downstream exon. Frequency plots were then calculated using the same method as for the junction-containing reads.

##### Detection of intron splicing-order

750,490,820 reads from two RNA-seq HeLa control samples (paired end, 51 nt, run IDs SRR514854 and SRR514855) were downloaded from the NCBI sequence read archive. These reads are assumed to be enriched in reads from partially spliced transcripts because they come from the rRNA-depleted, not polyA-selected nuclear RNA fraction. After the removal of the Illumina 3′ sequencing adaptor (AGATCGGAAGAGCACACGTCTGAACTCCAGTCA) with Cutadapt, the reads were mapped to the human genome assembly Hg19 with the splice mapper Hisat2 version 2.0.5. To enhance mapping of spliced reads, Hisat2 was supplied with two sources of splicing information. First, with the Hg19 Ensembl genome annotation (GRCh37.p13). Second, with specifically extracted splice junctions for all EJC-sensitive exons corresponding to the most common upstream and downstream introns. Last, evidence counts for the upstream or downstream intron spliced first were extracted from all mapped read pairs with a custom program and Samtools version 1.3.1. A read pair mapping to the upstream/downstream splice junction of an exon and to the downstream/upstream intron did account for one evidence count for the upstream/downstream intron spliced first. Only those exons where the sum of the two evidence counts for upstream/downstream intron spliced first was at least 10 were included in this analysis. Exons are binned in RS and nonRS categories if their RS-5ss score is above or below 5.52 respectively. EC-sensitive exons were defined as having a dPSI < −0.1 in HeLa cells after eIF4A3 KD, whereas EJC-insensitive exons were defined as having absolute dPSI values < 0.02.

##### eIF4A3 sensitive microexon identification

Exon-exon junctions were created for all exons sensitive to KDs of EJC components using the coordinates of the canonical, upstream and downstream exons (A, C1 and C2, respectively). For each exon A of at least 21 nucleotides, we identified potential 5′ splice sites with MaxEntScan scores of at least 5.52 ranging from 0 to 15 nucleotides from the C1A junction. This created a library of four types of sequences per sensitive exon: C1-C2, C1-A, A-C2 and exon skipping events leading to full skipping of RS-exons or 1-15nt long RS-linked microexons. The scores of potential 5′ splice sites were calculated employing the Maximum Entropy (MaxEnt) metrics (Yeo and Burge, 2004). Reads from RNA-seq data from eIF4A3 KD in HeLa cells (Wang et al., 2014) were trimmed to 50 nucleotides using sliding windows of 25 nucleotides and aligned to the human hg19 genome using bowtie v1.1.2 with -m 1 -v 2 options. Reads that did not map to the genome were then mapped to the library of junctions using bowtie v1.1.2 with the following parameters: -f -a -v 2. We corrected read counts by the number of potentially mappable position in the sequence for 50-nucleotides reads and requiring a minimum of 8-nucleotides being mapped at each side of the junction. Only those events with at least 10 reads support were considered. We defined a metric, Percent Usage of RS-5SS (PURS), to estimate the use of the RS-5ss, with respect to the “canonical” one using corrected read counts:

$$PURS=\frac{RS-n^{*}100}{\left(C1-E+E-C2\right)/2}$$

The difference between the PURS in the eIF4A3 KD and control was expressed as delta PURS (ΔPURS). To identify competing 5′ splice sites upon eIF4A3 KD, we selected RS-exons or RS-linked microexons with MaxEnt score of at least 5.52 and dPURS of at least 10.

##### Branchpoint inference

Lariats arising from RS-exon-dependent splicing were computationally inferred as previously described (Pineda and Bradley, 2018) from RNA-seq data obtained from Mercer et al. (2015) and Taggart et al. (2017) with the following modification: rather than using all possible pairings between annotated 5ss and 3ss as input to the branchpoint inference procedure, all possible pairings between the 5′ends of exons and 3ss were used.

### Quantification and Statistical Analysis

All experiments were done at a minimum of three independent replicates, unless specified. Data in quantifications shown in Supplemental Figures are expressed as mean ± standard deviation (SD). Statistical analysis was performed with Student’s t-Test using R version 3.3.1. Significant differences (P < 0.05) are indicated with a star (^∗^).

To test for differences in ΔPSI between groups of exons with similar RS-5ss score in each experiment, ΔPSI values for each exon category (alternative (15% < PSI < 85%), highly-included alternative (85% < PSI < 98%) and constitutive exons (PSI > 98%) were calculated by comparing two groups of exons, one above and one blow a threshold defined by the RS-5ss score. The RS-5ss score threshold produced two groups of exons which could be tested for skew in ΔPSI values using the Wilcox rank sum test. A range of RS-5ss score threshold values was then examined, from −20 to 8, in order to determine how the ΔPSI values were affected by the threshold. The signed P value (based on the sign of the test statistic and the generated P value) for each RS-5ss score threshold are shown in Figures 1G, 2A, and 2B.

### Data and Software Availability

The accession number for the iCLIP sequencing data files for PRPF8 iCLIP upon eIF4A3 KD reported in this paper is ArrayExpress: E-MTAB-7269, and for mRNAseq data of *Drosophila melanogaster* S2 cells after EJC component KD is ArrayExpress: E-MTAB-7271. Raw and processed iCLIP data are available also at https://imaps.genialis.com. The mRNaseq data of *Drosophila melanogaster* S2 cells after EJC component knockdown under E-MTAB-7271. Original gel and capillary electrophoresis images and quantification for all figures are available at https://doi.org/10.17632/7f9z9yxcx8.1.

#### Public RNA-seq data

ENCODE RNA-seq data for all 191 K562 cell line shRNA KD samples were downloaded from https://www.encodeproject.org (See experiments accession numbers in Table S7) and processed using the RNA-seq analysis pipeline outlined below. Other public RNA-seq data with the following accession numbers were downloaded from the short read archive https://www.ncbi.nlm.nih.gov/sra/ and processed using the RNA-seq analysis pipeline.

- Studies that included KD of at least one previously reported EJC-associated factor to identify factors that contribute to inclusion of RS-exons: Human: GEO: GSE63091, GSE52834, GSE73060, GSE81460, GSE86148 and mouse: GSE69733.

- RNaseq data from *Magoh* haploinsufficient mouse brain (*Magoh*^Mos2/+^) to analyze the effect of lower levels of EJC in the brain: GEO: GSE85576.

## Supplemental Information

Document S1. Figures S1–S7 and Tables S1–S4

Table S5. Sequences of the Splicing Reporters Used in this Study, Related to Figures 1, 3, 4, and 5

Table S6. Genomic Coordinates of High-Confidence RS-Lariats, Related to Figure 6

Table S7. Accession Numbers for RNA-Seq Data from the ENCODE Consortium Used in Figure 2B

Document S2. Article plus Supplemental Information
