# Supplementary material for: Exon Junction Complex Shapes the Transcriptome by Repressing Recursive Splicing
Source: Mol Cell. 2018 Nov 1;72(3):496–509.e9. doi: 10.1016/j.molcel.2018.09.033 (PMC6224609; doi:10.1016/j.molcel.2018.09.033)
Supplement: Document S1. Figures S1–S7 and Tables S1–S4 [file mmc1.pdf]

Molecular Cell, Volume *72*

Supplemental Information

Exon Junction Complex Shapes

the Transcriptome by Repressing Recursive Splicing

Lorea Blazquez, Warren Emmett, Rupert Faraway, Jose Mario Bello Pineda, Simon Bajew, Andre Gohr, Nejc Haberman, Christopher R. Sibley, Robert K. Bradley, Manuel Irimia, and Jernej Ule

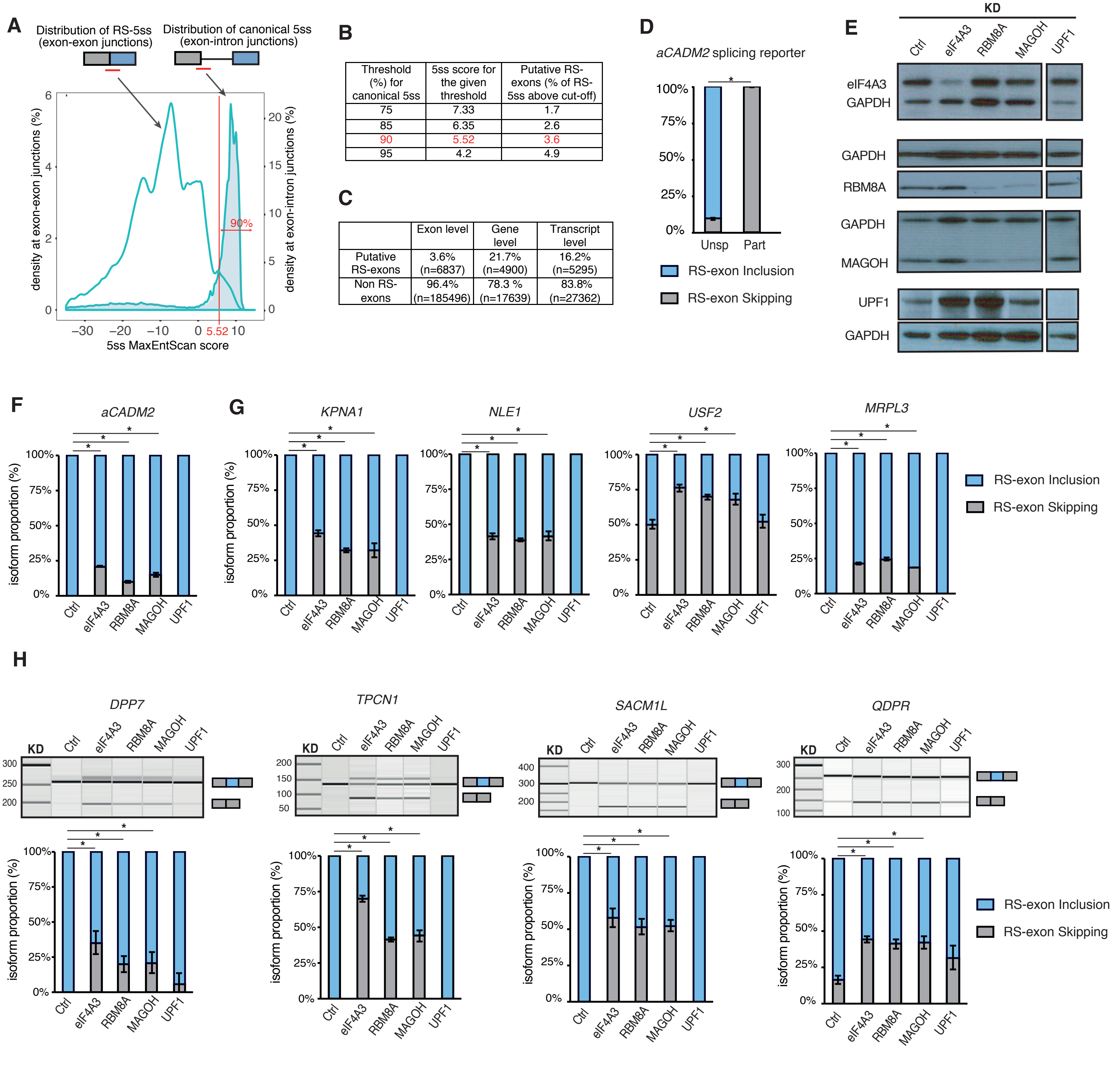

B

Threshold (%) for canonical 5ss	5ss score for the given threshold	Putative RS-exons (% of RS-5ss above cut-off)
75	7.33	1.7
85	6.35	2.6
90	5.52	3.6
95	4.2	4.9

C

	Exon level	Gene level	Transcript level
Putative RS-exons	3.6% (n=6837)	21.7% (n=4900)	16.2% (n=5295)
Non RS-exons	96.4% (n=185496)	78.3 % (n=17639)	83.8% (n=27362)

Figure S1. Related to Figure 1: Core EJC components promote inclusion of putative 'RS-exons', which are abundant in the transcriptome
A. Distribution of annotated exons according to their canonical 5ss MaxEntScan score (filled) or their RS-5ss at preceding exon-exon junction (line). The red line denotes the threshold above which 90% of all canonical 5ss are located and the number in red is the 5ss cut-off value associated with that threshold. **B.** Table showing the 5ss MaxEntScan scores associated with different thresholds and the proportion of putative RS-exons according to that 5ss cut-off value. In red, the threshold selected to quantify "putative RS-exons". **C.** Table showing the proportion of RS-exons at exon, gene and transcript level according to ENSEMBL GCRh37 annotation. **D.** Quantification of RT-PCR experiments shown in Figure 1C. **E.** Western-blot validation of eIF4A3, RBM8A, MAGOH and UPF1 protein depletion after siRNA transfection in HeLa cells. GAPDH is used as an input control. UPF1 KD sample was run in the same gel, but it is shown as a separate box as there were other lanes in between that are not shown. **F.** Quantification of RT-PCR experiments shown in Figure 1D. **G.** Quantification of RT-PCR experiments shown in Figure 1G **H.** RT-PCR validation and quantification of RS-exon skipping events in 4 different genes after KD of EJC core factors or UPF1 in HeLa cells (n=3, 3 independent experiments). For figures B, D, E and F, blue and grey columns indicate the percentage of RS-exon inclusion and skipping isoforms respectively. Data are mean ± standard deviation. *P<0.05.

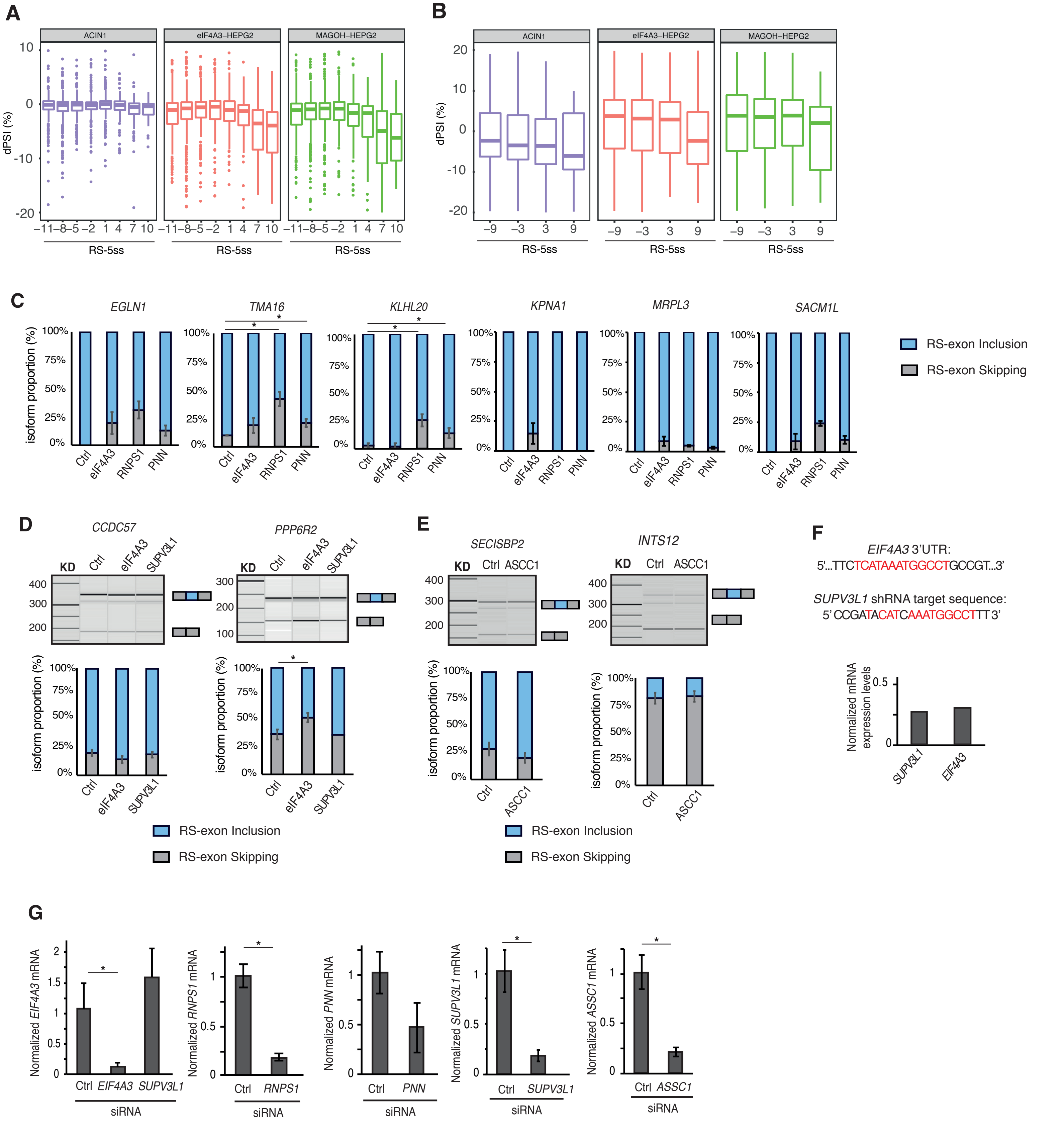

Figure S2. Related to Figure 2: PNN and RNPS1 contribute to the inclusion of 'annotated RS-exons'

A. Box plots representing the difference in percentage spliced in (dPSI) in highly included exons (PSI>90%) after knocking down different RBPs. Exons are binned by their RS-5ss score and dPSI for each bin is calculated by comparing its average PSI in each knockdown experiment to the control experiment.The RS-5ss values in the x-axis indicate the midpoint of each group. Negative dPSI values indicate exon skipping. **B.** Same as A but for alternative exons with PSI < 90%. **C.** Quantification of RT-PCR experiments shown in Figure 2E (n=3, 3 independent experiments, except for *EGLN1, KPNA1, MRPL3* and *SACM1L*, where n=2). **D-E.** RT-PCR Qiaxcel images (top) and quantification (down) of RS-exon skipping events upon *SUPV3L1* (D) or *ASCC1* (E) KD. eIF4A3 KD was included when *SUPV3L1* was knocked-down for comparison (n=3, 3 independent experiments). For quantification of RT-PCR experiments shown in C-E, blue and grey columns indicate RS-exon inclusion and skipping respectively. Data are mean ± standard deviation. *P<0.05. **F.** Diagram indicating *SUPV3L1* shRNA target sequence and *EIF4A3* 3'UTR sequence. The nucleotides shared in both sequences are indicated in red. Below, quantification of *SUPV3L1* and *EIF4A3* mRNA levels in SUPV3L1 KD ENCODE RNA-seq data showing that SUPV3L1 KD leads to a decrease in *EIF4A3* expression level. **G.** Quantification of *EIF4A3*, *RNPS1, PNN*, *SUPV3L1* and *ASCC1* mRNA levels by qPCR after their respective KD using gene-specific siRNAs (n=3, 3 independent experiments).

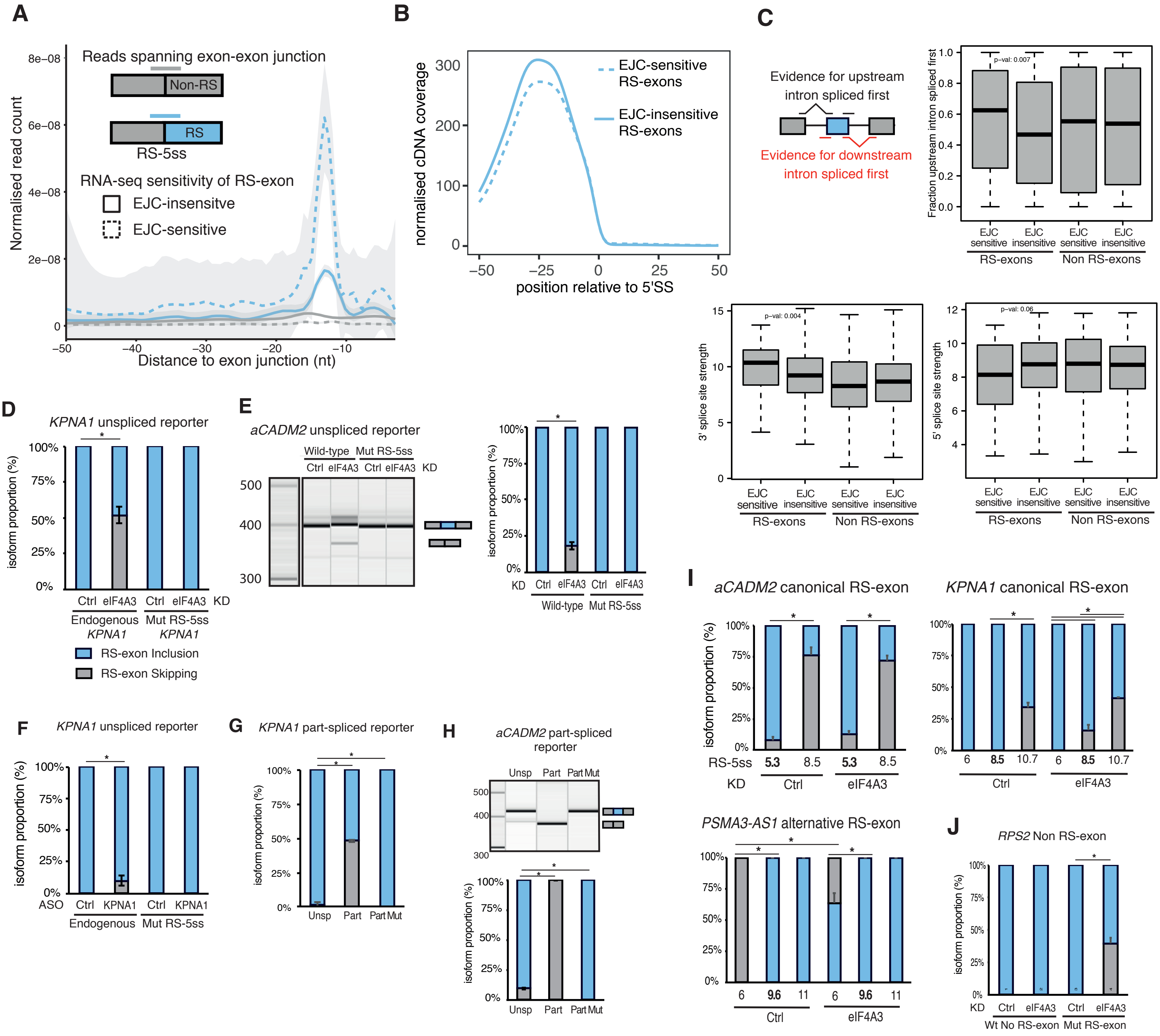

Figure S3. Related to Figure 3: Skipping of annotated RS-exons upon EJC KD results from recursive splicing
A. Metaprofile of the distance between read starts and exon-exon junctions from PRPF8 iCLIP in HeLa cells treated with eIF4A3 siRNA (n=4, 2 independent experiments). Reads that span exon-exon junctions at the 5' end of an RS-exon or non RS-exon are plotted in blue or gray respectively. Read counts have been normalised by the total number of crosslinks for each sample and the total number of exons in each class (RS sensitive = 890, RS insensitive = 3,741, non-RS sensitive = 14,486, non-RS insensitive = 115,924).The data was smoothed using LOESS with a span of 0.2. Shaded regions represent 95% confidence intervals. Exons containing only one read across all 8 samples were excluded from the analysis. **B.** Density plot showing the normalised cDNA coverage of eIF4A3 and BTZ iCLIP crosslinking around the 5ss upstream of RS-exons that are EJC-sensitive (green line, ΔPSI<-0.15 in KD of eIF4A3, RBM8A and/or CASC3) and EJC-insensitive (gray line, remaining RS-exons with >10 junction reads) in HeLa cells. **C**. Top left: Diagram showing the reads from RNAseq data used to analyse intron splicing order. Top right and below: Box plots showing the fraction of reads supporting co-transcriptional splicing and 3ss and 5ss scores in 4 different categories of highly included exons (PSI > 85). RS-exons are classified as having a RS-5ss MaxEntScan score of 5.52 or higher. EJC sensitive exons have a dPSI<-0.1 upon eIF4A3 knockdown whereas EJC insensitive exons are -0.02<dPSI< 0.02. **D.** Quantification of RT-PCR experiments shown in Figure 3C. **E.** Left: Qiaxcel analysis of endogenous or mutant *aCADM2* RS-exon splicing pattern after eIF4A3 KD in HeLa Flp-In cells which stably express wild-type or mutant RS-5SS *aCADM2* splicing reporters (n=3, 3 independent experiments). Right: Quantification of RT-PCR experiments shown in the left. **F.** Quantification of RT-PCR experiments shown in Figure 3D. **G.** Quantification of RT-PCR experiments shown in Figure 3E. **H.** Top: Qiaxcel analysis of *aCADM2* RS-exon splicing pattern after transient transfection of unspliced (Unsp), part-spliced (Part) and part-spliced mutant (Part Mut) plasmids in HeLa cells (n=3, 3 independent experiments). Bottom: Quantification of RT-PCR experiments shown on top. **I.** Quantification of RT-PCR experiments shown in Figure 3F. **J.** Quantification of RT-PCR experiments shown in Figure 3H. For quantification images shown in B to H, blue and grey columns indicate the percentage of RS-exon inclusion and skipping isoforms respectively. Data are mean ± standard deviation. *P<0.05.

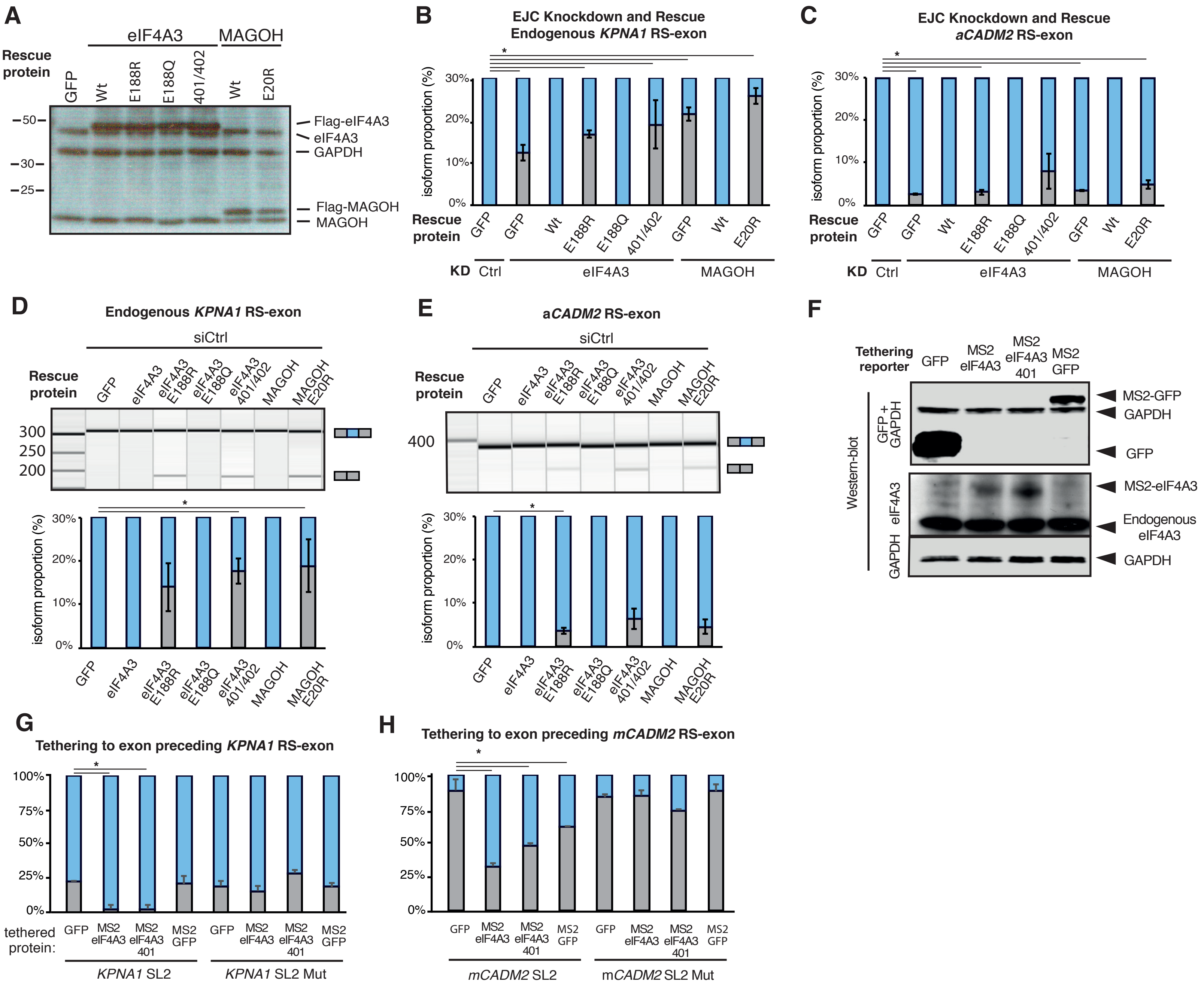

Figure S4. Related to Figure 4: Stable EJC deposition is required to block recursive splicing

A. Western-blot of eIF4A3 and MAGOH proteins to validate the expression of siRNA resistant Flag-tagged EJC components in HeLa cells. GAPDH is used as an input control. **B-C.** Quantification of *KPNA1* (B) and *aCADM2* (C) RS-exon splicing shown in Figure 4A (n=3, 3 independent experiments). **D-E.** Qiaxcel analysis (up) and quantification (down) of *KPNA1* (D) or a*CADM2* (E) RS-exon splicing pattern after overexpression of wild-type or mutant Flag-tagged EJC components in a control siRNA background (n=3, 3 independent experiments). **F.** Western-blot of MS2-rescue proteins used in tethering experiments.GAPDH is used as an input control. **G-H.** Quantification of *KPNA1* (G) and *mCADM2* (H) RS-exon splicing shown in Figure 4B (n=3, 3 independent experiments). For quantification images shown in B to E and G-H, blue and grey columns indicate RS-exon inclusion and skipping respectively. Data are mean ± standard deviation. *P<0.05.

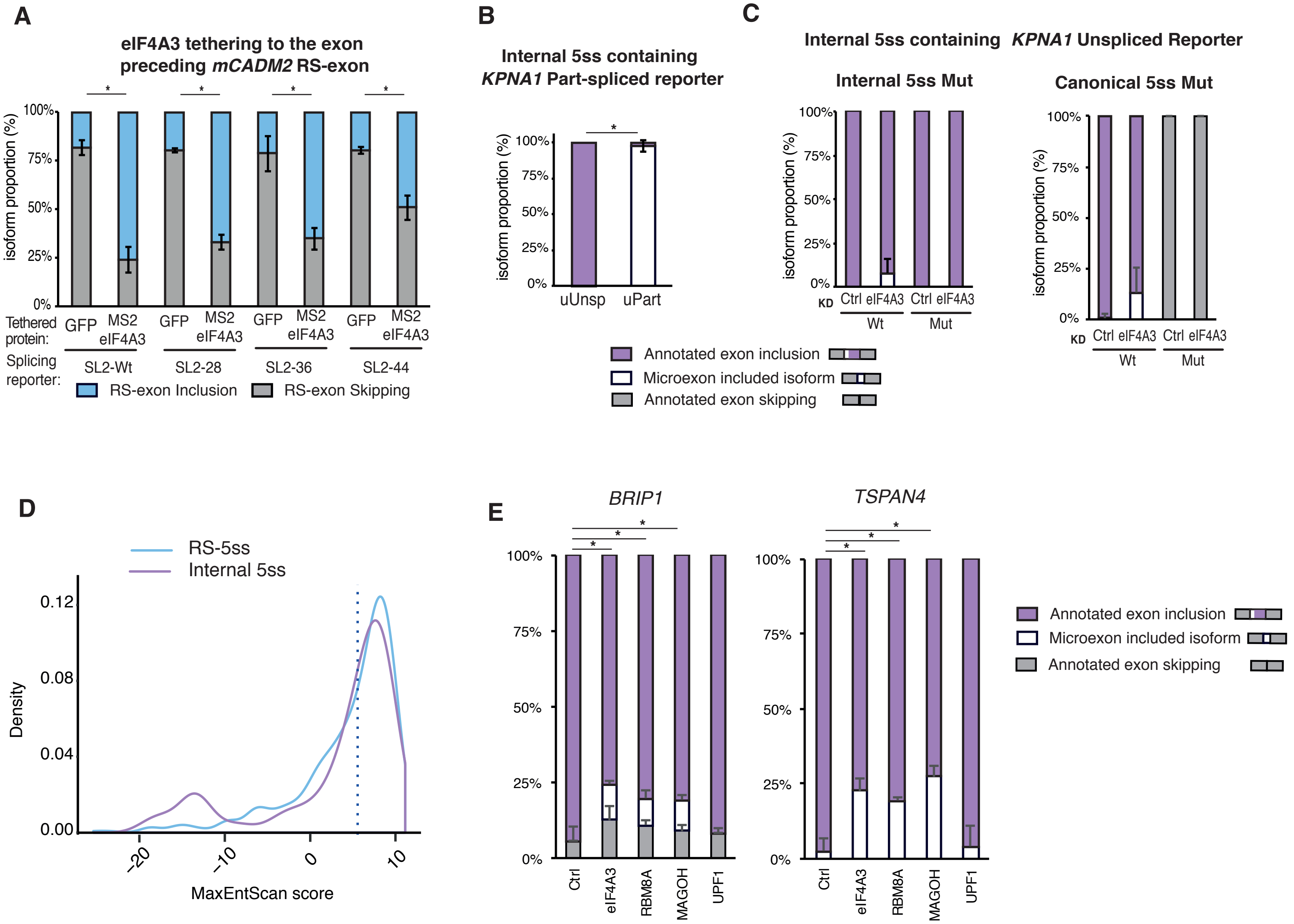

Figure S5. Related to Figure 5: EJC depletion leads to inclusion of cryptic microexons

A Quantification of RT-PCR experiments shown in Figure 5A. Blue and grey columns indicate the percentage of RS-exon inclusion and skipping isoforms respectively. Data are mean ± standard deviation. *P<0.05. **B** Quantification of RT-PCR experiments shown in Figure 5B. Purple and white columns indicate inclusion of annotated exon or microexon containing isoform respectively. Data are mean ± standard deviation. *P<0.05. **C** Quantification of RT-PCR experiments shown in Figure 5C. Purple and white columns indicate inclusion of annotated exon or microexon containing isoform respectively. Data are mean ± standard deviation. *P<0.05. **D** Density plot showing the distribution of RS-5ss MaxEntScan scores for RS-exons and cryptic 5ss MaxEntScan scores for cryptic microexons with dPURS >= 10 in HeLa cells. **E** Quantification of Qiaxcel images shown in Figure 5E. Purple and white columns indicate inclusion of annotated exon or microexon containing isoforms respectively. Grey columns in *BRIP1* represent annotated exon skipping isoform. Data are mean ± standard deviation. *P<0.05.

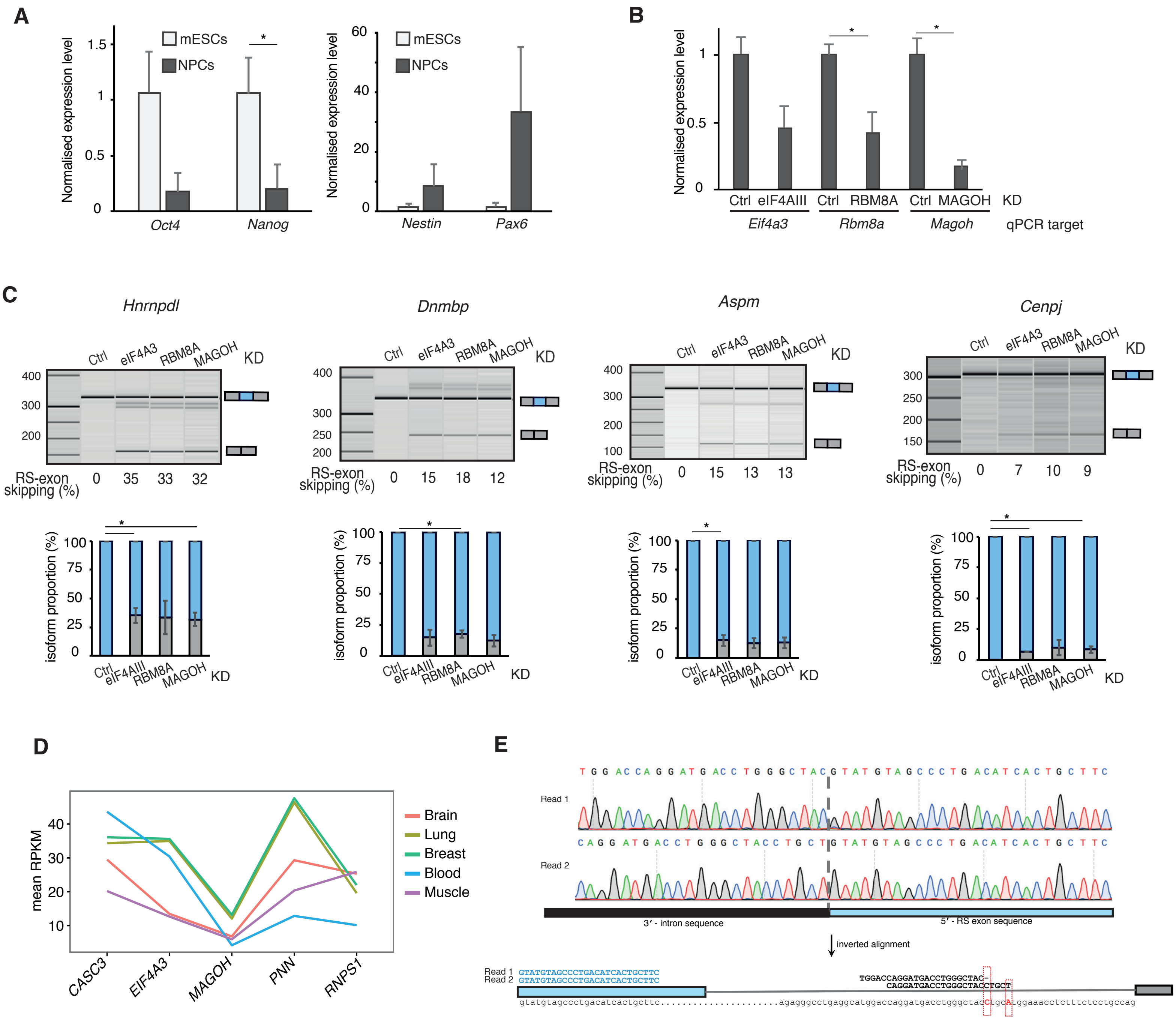

Figure S6. Related to Figure 6: EJC-mediated repression of Recursive splicing in the brain and physiologic alternative splicing of RS-exons
A. Quantification of the expression level of mESC (Oct4 and Nanog) and NPC (Nestin and Pax6) specific genes by qRT-PCR (n=3, 3 independent experiments). Data are mean ± standard deviation. *P<0.05. **B.** Quantification of Eif4a3, Rbm8a and Magoh mRNA levels by qPCR after their respective KD using shRNAs (n=3, 3 independent experiments). Data are mean ± standard deviation. *P<0.05. **C.** Top: Qiaxcel RT-PCR validation of RS-exon skipping events in neuronal precursor cells (NPCs) derived from mouse embryonic stem cells (mESCs) after depletion of core EJC components (n=3, 3 independent experiments). Bottom: Quantification of RT-PCR experiments shown in Figure 6C. Blue and grey columns indicate RS-exon inclusion and skipping respectively. Data are mean ± standard deviation. *P<0.05. **D.** Line graph depicting the mean RPKM of EJC factors across 5 tissues as defined by the GTEx RNA-seq data. **E.** Chromatograms of Sanger-sequencing results showing DNA sequences supporting RS-lariats. Two different sequences supporting the usage of alternative branchpoints were obtained.

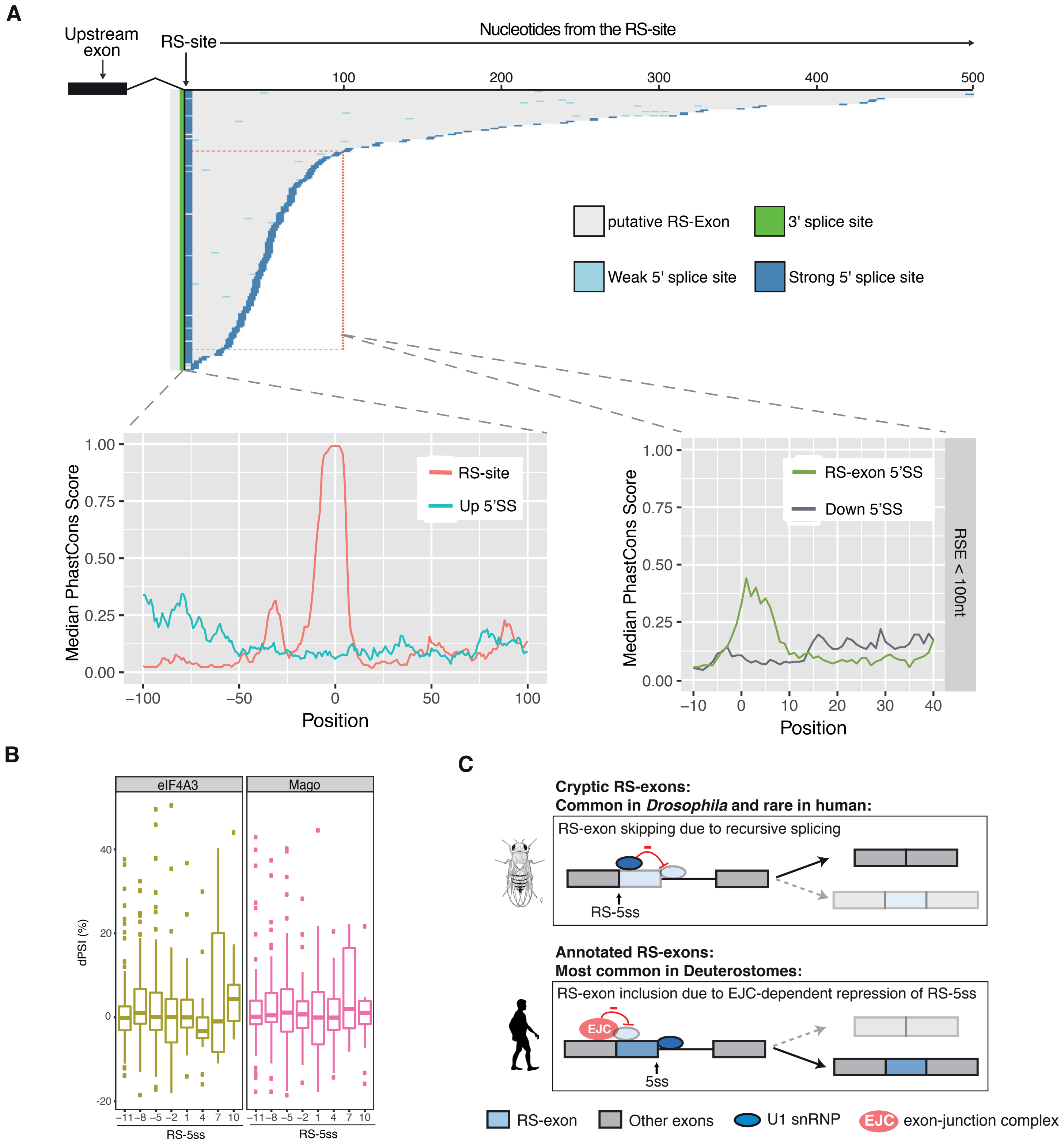

Figure S7: Related to Figure 7: Analysis of RS-exons inclusion across evolution

A. Top: Graphical representation of the RS-sites previously annotated in *D. melanogaster* and the nearest 5ss present downstream of them. The sequence between both sites is considered a putative RS-exon. Bottom: Sequence conservation analysis of the region downstream the RS-sites that contain a strong 5ss sequence within a 100nt window. Conservation strength is shown in red for RS-sites, blue for the nearest upstream 5ss (Up 5ss), green for the nearest downstream 5ss (RS-exon 5ss) and grey for further downstream 5ss (Down 5ss). **B.** Box plots representing the difference in percentage-spliced-in (dPSI) of exons after knocking down eIF4A3 or Mago in Drosophila S2 cells. Exons are binned by their RS-5ss score and dPSI for each bin is calculated by comparing its average PSI in each knockdown experiment to the control experiment.The RS-5ss values in the x-axis indicate the midpoint of each group. Negative dPSI values indicate exon skipping. **C.** RS-exon skipping due to recursive splicing, which leads to cryptic RS-exons, appears to be common in *Drosophila* but rare in human, whereas the proportion of canonical RS-exons is increased in Deuterostomes.

**Table S1: Target site sequences of siRNAs, Related to STAR Methods**	
**Gene name**	**Sequence**
MAGOH	CGGGAAGUUAAGAUAUGCCA
MAGOHB	CAGGCUGUUUGUAUAUUUAAU
EIF4A3-3UTR	GCAGCAGATCAGTGGGATGAG
MAGOH-3UTR	CTGAATATTGGTGTGGACA
SUPV3L1	CCGATACATCAAATGGCCTTT
ASCC1	GAATCATTTGATGGCCGAAAT
RNPS1	GCATCCAGCCGCTCAGGAA
PNN	GGTAAGGTGGCTCAGCGAG
RBM8A	CGCTCTGTTGAAGGCTGGA
CASC3	GATCGGAAGAATCCAGCAT

Table S2: Oligonucleotides sequences used for cloning, Related to STAR Methods	
**Name**	**Sequence**
**PSMA3 Fw-I**	ACTCGAAAGCTTAGCTTGTCGGCGCCATTTTG
**PSMA3 Rv-I**	ATCGATTGGATCCGGTTACGAAGTTTACCATGGCTGCCTG
**PSMA3 Fw-II**	CGTAACCGGATCCAATCGATATCGCAGTCTTTAAGTTACA
**PSMA3 Rv-II**	ATCGATTGAATTCGGTTACGGTCTTATCAGGCTACAAAAG
**PSMA3 Fw-III**	CGTAACCGAATTCAATCGATAGCAAATTTGTGTGTGTGGT
**PSMA3 Rv-III**	ACTCGAGCGGCCGCCACAAACAGATCGCAGATCC
**PSMA3_6_Fw**	TGTGTAGCAAAATGTTTCACTGTACAAATTCCCACCTAAAGTGAAAAAAAAAAAAAAAAGTG
**PSMA3_6_Rv**	CACTTTTTTTTTTTTTTTCACTTTAGGTGGGAATTTGTACAGTGAAACATTTTGCTACACA
**PSMA3_11_Fw**	CCACTTTTTTTTTTTTTTTCACTTTAGGTAAGTATTTGTACAGTGAAACATTTTGCTACACA
**PSMA3_11_Rv**	TGTGTAGCAAAATGTTTCACTGTACAAATACTTACCTAAAGTGAAAAAAAAAAAAAAAAGTGG
**aCADM2_8.5_Fw**	CTGATTACACTGGAATGATTAATGTACTTACCTAAAACAGAAAGAAATAAAACA
**aCADM2_8.5_Rv**	TGTTTTATTTCTTTCTGTTTTAGGTAAGTACATTAATCATTCCAGTGTAATCAG
**KPNA1_6_Fw**	ACTCTGCAGAGCTGAGCAATTCAGCATCACCTAAAAGAAAAAGTTTGAAAAT
**KPNA1_6_Rv**	ATTTTCAAACTTTTTCTTTTAGGTGATGCTGAATTGCTCAGCTCTGCAGAGT
**KPNA1_10.7_Fw**	TAAAATTTTCAAACTTTTTCTTTTAGGTAAGACTGAATTGCTCAGCTCTGCAGAG
**KPNA1_10.7_Rv**	CTCTGCAGAGCTGAGCAATTCAGTCTTACCTAAAAGAAAAAGTTTGAAAATTTTA
**HindIII_MainCADM2_1444Fw**	ACTCGAAAGCTTCTGCTGCCGCCGATCCGAGT
**CADM2_Main_SL2_Rv**	GGCACGGCTGATGCTCGTGCTTTGGAGAACGGCGCTGCGTTTC
**CADM2_Main_SL2-Int_Fw**	AGCACGAGCATCAGCCGTGCCTCGCTACAAGGTAATCCCCGCC
**p5_2364NotI_Rv**	ACTCGAGCGGCCGCCAGTGTGATGGATATCTGCA
**KPNA1 MS2-Mut_Fw**	GGCACGGCTCATGCTCGTGCTTTTCCC
**KPNA1 MS2-Mut_Rv**	GGGAAAAGCACGAGCATGAGCCGTGCC
**CADM2_SL2Mut_Fw**	GGCACGGCTCATGCTCGTGCTTTGGAG
**CADM2_SL2Mut_Rv**	CTCCAAAGCACGAGCATGAGCCGTGCC
**E188Q Fw**	ACGTGCTATCAAAATGTTGGTTTTGGATCAAGCTGATGAAATGT
**E188Q Rv**	ACATTTCATCAGCTTGATCCAAAACCAACATTTTGATAGCACGT
**GFP_Fw_XhoI**	ACTCGACTCGAGATGGTGAGCAAGGGCGAGGA

**GFP_Rv_NotI**	ACTCGAGCGGCCGCTCACTTGTACAGCTCGTCCATGC
**mMagoh shRNA Fw**	CCGGGCAAGTTCGGTCATGAGTTCCAATCTCGAGATTGGAACTCATGACCGAACTTGCTTTTTG
**mMagoh shRNA Rv**	AATTCAAAAAGCAAGTTCGGTCATGAGTTCCAATCTCGAGATTGGAACTCATGACCGAACTTGC
**mRbm8a shRNA Fw**	CCGGGCGGACCTTGTGTTTATATTTAATCTCGAGATTAAATATAAACACAAGGTCCGCTTTTTG
**mRbm8a shRNA Rv**	AATTCAAAAAGCGGACCTTGTGTTTATATTTAATCTCGAGATTAAATATAAACACAAGGTCCGC
**meIf4a3 shRNA Fw**	CCGGGAGGAGGACATGACCAAAGAATCTCGAGATTCTTTGGTCATGTCCTCCTCTTTTTG
**meIf4a3 shRNA Rv**	AATTCAAAAAGAGGAGGACATGACCAAAGAATCTCGAGATTCTTTGGTCATGTCCTCCTC
**mControl shRNA Fw**	CCGGCGGCTGAAACAAGAGTTGGAATCTCGAGATTCCAACTCTTGTTTCAGCCGTTTTTG
**mControl shRNA Rv**	AATTCAAAAACGGCTGAAACAAGAGTTGGAATCTCGAGATTCCAACTCTTGTTTCAGCCG

Table S3: Oligonucleotides sequences used for PCR/RT-PCR, Related to STAR Methods	
**Name**	**Sequence**
**KPNA1 Exon 10 Fw**	AGTGGTTTCTCCTGCTTTGC
**KPNA1 Exon 12 Rv**	GCTGATCCTCCAGAAGTTGC
**KPNA1 Exon13 Rv**	CAGAGCGGCTTGATACAACC
**BGHpA Rv**	TTAGGAAAGGACAGTGGGAG
**aCADM2 Fw**	GCTGTCACCTCTCAATATCACAAG
**mCADM2 Fw**	TTCCTTCCCCAGCCCTTTAG
**USF2 Fw**	GCGACCACAACATCCAGTAC
**USF2 Rv**	ACACTGGACGCTGGGAAATA
**DPP7 Fw**	TGTCAGACGAGAAGGACCTG
**DPP7 Rv**	GTAGAGCCGGTAGATGTCGT
**NLE1 Fw**	ACTGAAGGTGTGGGATGTGA
**NLE1 Rv**	CAGCCTTTGTTCTCTGGCAG
**MRPL3 Fw**	CCAAGGATGGTCAAAAGCAT
**MRPL3 Rv**	GCCTGGTTTAATTGCAGCT
**TPCN1 Fw**	TTTGATGAGCTTCCCAGGAC
**TPCN1 Rv**	GTCTCCACGAGGATCCAGAC
**SACM1L Fw**	TCCTCCTTCAGCTGTCACAA
**SACM1L Rv**	CGCTGCAAAGTATGGGTCAA
**QDPR Fw**	ATCGACCATCTCCAGCCATC
**QDPR Rv**	GCATTGATTTCCTGTTCATCGG
**TMA16_Fw**	TCAAAGAAAGATGCTTGTGAACT
**TMA16_Rv**	TTTCCTCTTGCACGTCTTGG
**KLHL20_Fw**	GTTGGTGCAGTGGAGATGC
**KLHL20_Rv**	AGAACCCTCCTAACACAGCC
**RAD7_Fw**	TATAGAGGAAAGGGGCCAAGA
**RAD7_Rv**	GGTTGCCTTTCTAAAACTTGAGC
**EMG1_Fw**	CAGTGTTCGAGCAGCTGATG
**EMG1_Rv**	AAGTTTTGCACAGGTGAGGG
**CCDC57_Fw**	TGCCTGGGATGCTCAAATTG
**CCDC57_Rv**	CCACCTCCTGCTCCTGTAG
**GANC_Fw**	CAGCACCAGGTTCCAAATCA
**GANC_Rv**	CCTGTTCCTGGCCATTTCTG
**PPR6R2_Fw**	GAGAGCTTCGTGGAGGAGAC
**PPR6R2_Rv**	TGATGTTGTCGTCCTGGTCG
**SECISBP2L_Fw**	TTGGTCCAATGTAACTTGC
**SECISBP2L_Rv**	TCTCATCCTTAGCATTTCCA
**INTS12_Fw**	CGGGAACAGACGGATCGG

**INTS12_Rv**	AACTGGAATCAATGCCCCGA
**mHnrnpdl_Fw**	GCAACAACAGAAAGGAGGCA
**mHnrnpdl_Fw**	CCCAGCGTCCTCCTTTAGTA
**mAspm_Fw**	CCCTTGGGTTGCTTTGGAAA
**mAspm_Rv**	ACGGGTGGTAATGGTGGATC
**mDnmbp_Fw**	TGGAGAAGAGAGCCAAGGTG
**mDnmbp_Fw**	AGGTGCTTCTGGGTCTTCTC
**mFlna_Fw**	TGGGACTCCTGGGATGCTA
**mFlna_Fw**	CTTCTCCCTGTCCAGCACTT
**RPS2_Fw**	GATTATGCCAGTGCAGAAGC
**TSPAN4_Fw**	TCCTCTTCTTCGCCTACACG
**TSPAN4_Rv**	AAGCAGCAGGAGTCAGGTAC
**HSPD1_Fw**	CAGTCCATTGTACCTGCTCTTG
**HSPD1_Rv**	AAGTCATGAGGCTGAACGTC
**BRIP1_Fw**	ACACAGAAATGATTGGGGAGC
**BRIP1_Rv**	TTGTTTGTTGAAAGTTGGGCT
**RPL18A_Fw**	GACACTTCCTTTTGCGGGTG
**RPL18A_Rv**	ATTCCCGGTACATGTTGTGG
**AP1G2-RS-outer_Fw**	AGGGGACTAAGGGGGACAG
**AP1G2-RS-outer_Rv**	CTGAGGGAGGCATCAGTTTC
**AP1G2-RS-inner_Fw**	GACAGGTCCCTGGGGAAG
**AP1G2-RS-inner_Rv**	CTGAGGGAGGCATCAGTTTC
**AP1G2-Std-outer_Fw**	GCAGTGATAGAGGGGACAGG
**AP1G2-Std-outer_Rv**	TCAGACTGCACCAGTCGAAG
**AP1G2-Std-inner_Fw**	GCAGTGATAGAGGGGACAGG
**AP1G2-Std-inner_Rv**	CAGGAGGCTCTGGAGGAAG
**AP1G2-upstream of RSE-outer_Fw**	TCCCTATACCCAGGACAGGAC
**AP1G2-upstream of RSE-outer_Rv**	CAGGAGGCTCTGGAGGAAG
**AP1G2-upstream of RSE-inner_Fw**	TCCCTATACCCAGGACAGGAC
**AP1G2-upstream of RSE-inner_Rv**	GAGGCTCTGGAGGAAGGAG
**AP1G2-downstream of RSE-outer_Fw**	GACAGGTCCCTGGGGAAG
**AP1G2-downstream of RSE-outer_Rv**	TAAACTGGCTCGAGTCTTGG
**AP1G2-downstream of RSE-inner_Fw**	ACCAGGATGACCTGGGCTAC
**AP1G2-downstream of RSE-inner_Rv**	TAAACTGGCTCGAGTCTTGG
**mago_dsRNA_Fw**	taatacgactcactatagggCCGTGTGTTTTCCCATCTCT
**mago_dsRNA_Rv**	taatacgactcactatagggGCCCTCGGGATCTTTTGAC
**eIF4AIII_dsRNA_Fw**	taatacgactcactatagggCCACCTTCTCCATCTCCATC
**eIF4AIII_dsRNA_Rv**	taatacgactcactatagggGCGACGAAGAACTGCTTGAT

Table S4: Oligonucleotides sequences used for qPCR, Related to STAR Methods	
**Name**	**Sequence**
**EIF4A3_qPCR_Fw**	ATGGCGACCACGGCCACGAT
**EIF4A3_qPCR_Rv**	GTGTCGAACGTGGGGGTCAC
**RNPS1_qPCR_Fw**	GGACAAAACCCGAAAGAGGC
**RNPS1_qPCR_Rv**	CCTCCTGTTGTCGTGTCTGC
**PNN_qPCR_Fw**	ACAAGAATCCACTGTTGCTACTG
**PNN_qPCR_Rv**	CAAAAGCCGCAGTTCTGTCT
**SUPV3L1_qPCR_Fw**	TGCAGTCTCATTCCCTGGAT
**SUPV3L1_qPCR_Rv**	ATTATCTTCCGCTGCATGGC
**ASCC1_qPCR_Fw**	GAAGTGGAGATGGCAGGGAT
**ASCC1_qPCR_Rv**	GAAAACGTTCCAGCACTCGA
**GAPDH_qPCR_Fw**	AAGGTGAAGGTCGGAGTCAA
**GAPDH_qPCR_Rv**	AATGAAGGGGTCATTGATGG
**mEif4a3_Fw**	TTTCACTGTGTCGTCCATGC
**mEif4a3_Rv**	TGTTGGGCAGGTCGTAGTTA
**mRbm8a_Fw**	GTCACTGGAGTCCACGAAGA
**mRbm8a_Rv**	GGTCCACGAACAAAACACCA
**mMagoh_Fw**	ACAAAGGCAAGTTCGGTCAT
**mMagoh_Rv**	GGCCACAGAGCATCATCTTC
**mOct4_Fw**	CTTCACCACACTCTACTC
**mOct4_Rv**	CCAGGTTCTCTTGTCTAC
**mNanog_Fw**	TGAGCTATAAGCAGGTTAAGAC
**mNanog_Rv**	CAATGGATGCTGGGATACTC
**mNestin_Fw**	GTCTCAGGACAGTGCTGAGCCTTC
**mNestin_Rv**	TCCCCTGAGGACCAGGAGTCTC
**mPax6_Fw**	GCGGAGTTATGATACCTACACC
**mPax6_Rv**	GAAATGAGTCCTGTTGAAGTGG
**mGAPDH_Fw**	AAGGGCTCATGACCACAGTC
**mGAPDH_Rv**	GGATGACCTTGCCCACAG
